# Kinetic Modeling of the Reversible or Irreversible Electrochemical Responses of FeFe-Hydrogenases

**DOI:** 10.1021/jacs.3c10693

**Published:** 2024-01-02

**Authors:** Andrea Fasano, Carole Baffert, Conrad Schumann, Gustav Berggren, James A. Birrell, Vincent Fourmond, Christophe Léger

**Affiliations:** † Laboratoire de Bioénergétique et Ingénierie des Protéines. CNRS, Aix Marseille Université, UMR, 7281 Marseille, France; ‡ Molecular Biomimetics, Department of Chemistry, Ångström Laboratory, Uppsala University, 75120 Uppsala, Sweden; § School of Life Sciences, University of Essex, Wivenhoe Park, Colchester CO4 3SQ, U.K.

## Abstract

The enzyme FeFe-hydrogenase catalyzes H_2_ evolution and oxidation at an active site that consists of a [4Fe-4S] cluster bridged to a [Fe_2_(CO)_3_(CN)_2_(azadithiolate)] subsite. Previous investigations of its mechanism were mostly conducted on a few “prototypical” FeFe-hydrogenases, such as that from ­(Cr HydA1), but atypical hydrogenases have recently been characterized in an effort to explore the diversity of this class of enzymes. We aim at understanding why prototypical hydrogenases are active in either direction of the reaction in response to a small deviation from equilibrium, whereas the homologous enzyme from (Tam HydS) shows activity only under conditions of very high driving force, a behavior that was referred to as “irreversible catalysis”. We follow up on previous spectroscopic studies and recent developments in the kinetic modeling of bidirectional reactions to investigate and compare the catalytic cycles of Cr HydA1 and Tam HydS under conditions of direct electron transfer with an electrode. We compare the hypothetical catalytic cycles described in the literature, and we show that the observed changes in catalytic activity as a function of potential, pH, and H_2_ concentration can be explained with the assumption that the same catalytic mechanism applies. This helps us identify which variations in properties of the catalytic intermediates give rise to the distinct “reversible” or “irreversible” catalytic behaviors.

## Graphical abstract

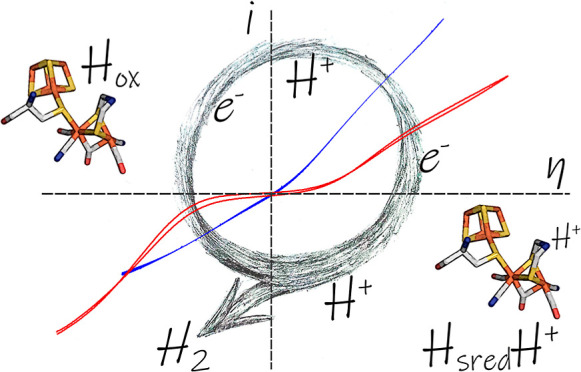

## Introduction

The redox reactions of H_2_ oxidation and production are catalyzed in Nature by metalloenzymes whose active sites are composed of abundant metals, Ni and Fe, and are called hydrogenases. Here, we focus on FeFe-hydrogenases, whose active site, called the “H-cluster”, is composed of a [4Fe-4S]_H_ cluster, linked by a cysteine to a dinuclear Fe center ([2Fe]_H_). The two Fe ions of [2Fe]_H_ are coordinated by three carbonyl ligands (one of which is bridging) and two cyanides and bridged by an amine-dithiolate (ADT) ligand. The Fe ion that is remote from the cubane is referred to as the “distal” Fe, or Fe_d_. In the catalytic cycle, H_2_ binds to the open coordination site on Fe_d_, and it is split heterolytically into a terminal hydride and a proton. The latter binds to the nitrogen of the ADT ligand,^1,2^ from where it is transferred to a nearby cysteine, and then further along a chain of acidic residues.^3,4^ This proton-transfer pathway is very conserved in the phylogenetic group of FeFe-hydrogenases that includes the most studied enzymes (called group “A”, or “prototypical” in ref 5). In these enzymes, long-range electron transfer may involve accessory FeS clusters.

Detailed information about the catalytic cycle has been obtained over decades of spectroscopic investigations. Some of the proposed catalytic intermediates are paramagnetic and hence detectable by electron paramagnetic resonance spectroscopy, but the spectroscopic technique that has become the most popular in hydrogenase research is Fourier transform infrared (FTIR) spectroscopy, which can detect the vibrations of the CO and CN diatomic ligands to identify various states of the H-cluster. Regarding prototypical hydrogenases, two distinct mechanistic hypotheses have emerged.^6^

The most oxidized catalytic intermediate is H_ox_, where the electronic configuration of the dinuclear cluster is Fe­(II)/Fe­(I) and the cubane is oxidized (2+). In model 1 (Figure 1D), the reduction of H_ox_ at high pH produces the H_red_ state, where the cubane is reduced. H_red_ has a p*K*_a_ value around 7, and at pH < p*K*_a_, the reduction of H_ox_ is coupled to a protonation, to give the H_red_H^+^ state. This protonation was evidenced by the pH dependence of the reduction potential of the H_ox_ state observed in spectroelectrochemical titrations of the active site at different pH values (the corresponding Pourbaix diagram is reproduced from ref 8 in Figure 1C). The vibrational band of the bridging CO ligand is apparently lost upon protonation, suggesting that the ligand shifts to a terminal position.^10,11^ However, recent data suggests that the band is only weak and highly broadened, as low-temperature measurements produce a more intense sharp band.^7,12,13^ It is hypothesized that binding of the proton to the nitrogen of the amine in the azadithiolate ligand induces an intramolecular electron transfer from the cubane to the dinuclear cluster, which becomes Fe­(I)/Fe­(I). An increased electron density at [2Fe]_H_ explains the larger red shift of the vibration bands in the H_ox_ to H_red_H^+^ transition than in H_ox_ to H_red_.^8^ Reduction of H_red_H^+^ gives the super-reduced state H_sred_H^+^,^14^ with a protonated amine and a reduced cubane, which is a tautomer of H_hyd_, with a hydride on Fe_d_.^15–18^ The second protonation of the doubly reduced state gives a series of elusive species, called H_hyd_H^+^ and H_ox_H_2_, from which the release of H_2_ completes the catalytic cycle by giving back H_ox_. Some spectroscopic signatures have been attributed to H_hyd_H^+^.^16,19^

**Figure 1:**
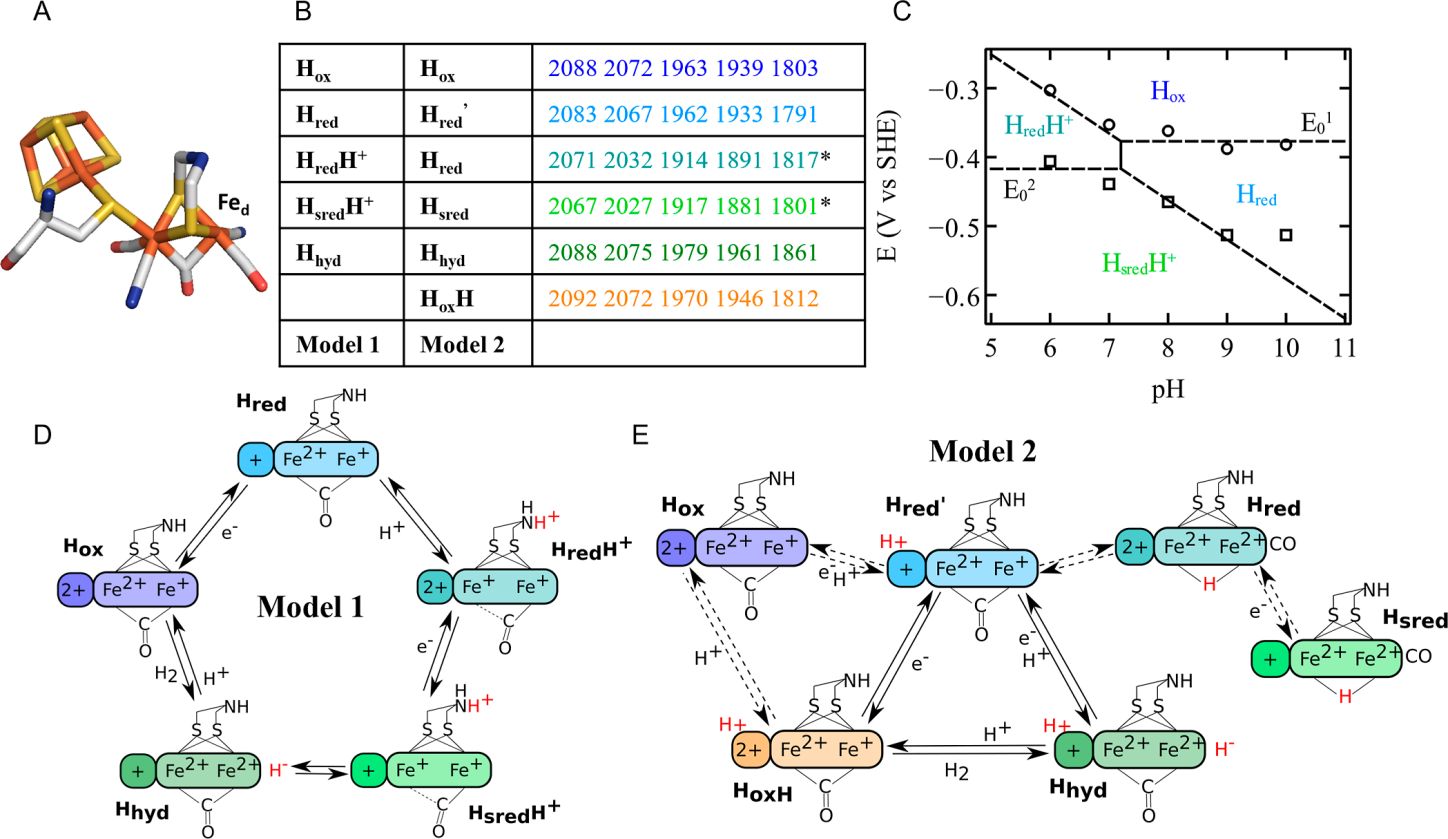
(A) Active site “H-cluster” of [FeFe]-hydrogenases (color code: Fe (orange), S (yellow), C (white), N (blue), and O (red)). (B) FTIR frequencies of the Cr HydA1 states in panels D and E; the states of models 1 and 2 that share the same FTIR signature are shown with the same color. Asterisks on the vibration frequencies of H_red_H^+^ and H_sred_H^+^ mark the bridging CO frequencies detected at low temperature in ref 7. (C) Pourbaix diagram of H_ox_, H_red_, H_red_H^+^, and H_sred_H^+^ transitions adapted from ref 8; (D) model 1 of the catalytic cycle of [FeFe]-hydrogenases, proposed in ref 8; and (E) model 2 of the catalytic cycle of [FeFe]-hydrogenases, proposed in ref 9.

In model 2 (Figure 1E), the FTIR signature assigned to the H_red_ state is instead attributed to a state called H_red_’ (panel E), and the H_ox_ to H_red_’ reaction supposedly involves the reduction and coupled protonation of the cubane. This was concluded from the pH dependence of the potential of the cubane, measured in enzyme variants where the nitrogen atom of the amine dithiolate bridge is replaced with a carbon atom.^20^ (However, this observation was recently challenged.^21^) Consistent with the above-described transformation between H_red_ and H_red_H^+^, H_red_’ is replaced at pH below 7 with the H_red_H^+^ state, which is called H_red_ in model 2 (Figure 5C in ref 9). However, at variance with model 1, it is assumed that the transition from H_red_’ to H_red_ at low pH is the result of a proton rearrangement, with a deprotonation of the cubane and the formation of a bridging hydride, and the bridging CO shifts to an apical position (something that is not supported by the spectroscopic investigations of Cr in ref 13, and not observed in any of the states of the H-cluster of hydrogenase I^12^). Considering the expected stability of this bridging hydride, the H_red_ species and the corresponding H_sred_ state obtained by further reduction (which has the same spectroscopic signature as the species denoted as H_sred_H^+^ in model 1) are considered to be off-pathway. Instead, reductive catalysis proceeds from H_red_’, whose one-electron one-proton reduction gives a hydride species also denoted as H_hyd_ but now considered protonated on the cubane in addition to Fe_d_. The so-called “regulatory” proton on the cubane is retained throughout the catalytic cycle. Further steps of reduction, protonation, and H_2_ release give back H_red_’ bypassing the H_ox_ state, which is also considered off-pathway (see Figure 2 of ref 9). Whether or not the spectroscopic signal assigned to H_ox_H reflects a true catalytic intermediate or an artifact is currently debated.^22,23^

Recently, two FeFe-hydrogenases whose active site environment differs significantly from that in group A have been characterized, although to a much lesser extent than for group A hydrogenases. These enzymes are (Tm) HydS^24^ and (Tam) HydS,^25^ from groups C and D, respectively.^26^ Albeit from two distinct phylogenetic groups, the latter enzymes feature an identical set of electron relay FeS clusters and are structurally very similar. Indeed, as compared to group A enzymes, most of the altered amino acids in the direct vicinity of the H-cluster are identical in *Tam* HydS and *Tm* HydS. In the sequences of both enzymes, the cysteine residue that accepts protons from the amine dithiolate ligand in group A hydrogenases is replaced with an alanine, and none of the residues that form the proton-transfer pathway in group A hydrogenases are present; a possible alternative pathway was recently identified in group D.^27^ The strong similarities between Tam HydS and Tm HydS suggest similar active site properties and function.

In Tm HydS, the H_ox_ FTIR signature is very similar to that of group A hydrogenases (compare Figure 1B and Figure 5A in ref 24), but the midpoint potentials of the two one-electron redox transitions are more separated than those of Cr HydA1 (−300 and −570 mV in Tm HydS^24^ vs −362 and −465 mV in Cr HydA1,^8^ both at pH 8), suggesting that the half-reduced state of the H-cluster is stable over a larger potential range in Tm HydS than in Cr HydA1. How this is determined by the environment of the H-cluster is unclear.^28^ Regarding Tam HydS, the H_ox_ state signature is again unremarkable, and, as is the case with Tm, the one-electron reduced state is very stable.^25^

In terms of functional properties, both Tam and Tm HydS have low activity (consistent with their putative role as H_2_ sensors), but Tam HydS is also remarkable in that catalysis is only observed upon application of a large driving force for H_2_ oxidation or evolution, and we assume that this is also the case for Tm HydS. This behavior, termed “irreversible catalysis”,^29–32^ is observed in voltammetric experiments where the enzyme undergoes direct electron transfer with an electrode and catalysis is detected as a positive or negative current (for H_2_ oxidation and evolution, respectively), as the electrode potential is shifted from the Nernst potential of the H^+^/H_2_ couple.^33,34^ In the particular case of Tam HydS, we ruled out the possibility that this irreversibility is the consequence of slow interfacial electron transfer or results from the catalytic cycle being different in the two directions of the reaction,^31^ but the relation between irreversible catalysis and the details of the catalytic cycle still needs to be established.

Here, we follow up on recent advances in the kinetic modeling of bidirectional catalytic voltammetry^35–40^ to propose an original point of view on the catalytic cycle of prototypical and atypical FeFe-hydrogenases. We aim at giving a full description of how the voltammogram shapes (catalytic potentials and limiting currents) depend on pH and H_2_ concentration. Our analysis of the voltammetry of Cr HydA1 appears to be fully consistent with model 1 and gives further information about the thermodynamic properties of the reduced catalytic intermediates. The Tam HydS voltammetric data can be analyzed assuming that the same catalytic mechanism is operational, and this helps us identify variations in properties of the catalytic intermediates that make the two enzymes behave “reversibly” and “irreversibly”.

## Results

### pH Titration of Cr HydA1

We recorded Cr HydA1 FTIR spectra at different pH values, from 5 to 10, at a constant H_2_ partial pressure (2% H_2_ in N_2_, *P*_tot_ = 1 atm), under equilibrium conditions. In this series of experiments, the enzyme was diluted from a concentrated stock solution into a buffer of the desired pH, so that the concentration of enzymes was always the same. Each experiment was performed in triplicate.

Figure 2A shows the spectral transition of Cr HydA1 from a high pH (blue) to a low pH (red). We have monitored the intensities of the bands of H_ox_ (1939 cm^–1^) and H_red_H^+^ (1891 cm^–1^), mostly present at low pH, and of H_red_ (1933 cm^–1^) and H_sred_H^+^ (1881 cm^–1^), mostly present at high pH.^8^ The H_hyd_ state was omitted because the corresponding band at around 1850 cm^–1^ was too small to be reliably quantified.

**Figure 2:**
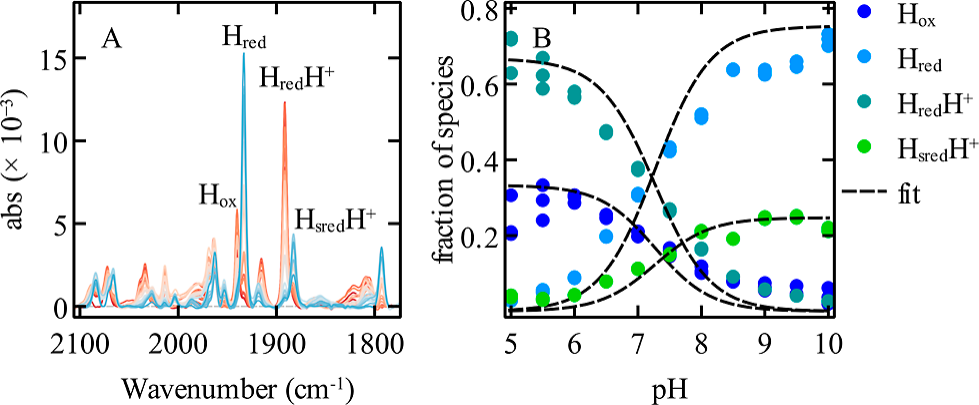
Cr HydA1 pH titration at 2% H_2_. (A) Overlay of the FTIR spectra baseline subtracted as a function of pH (from 5 (red) to 10 (blue)). (B) Fit of the fraction of states H_ox,_ H_red_, H_red_H^+^, and H_sred_H^+^ (same color code as in Figure 1) with the model proposed in ref 8 (Supporting Information Section S1.2), giving the following parameters: p*K* = 7.2, *E*_1_^0^ = −359 mV, and *E*_2_^0^ = −405 mV vs SHE. Each data point was measured in triplicate; the dispersion of the results is visible in panel B.

The sum of the intensities of the peaks corresponding to these four main states was constant across pH values (see Figure S1.1). We explain in Supporting Information Section S1.1 how we calculated the fractions of these species shown as a function of pH in Figure 2B, with the same color code as in Figure 1. By changing the pH at a constant H_2_ pressure, we also change the potential experienced by the enzyme at each pH value, as described by the Nernst equation. This allowed us to fit the model proposed in ref 8 to the data, considering two redox reactions, H_ox_/H_red_ and H_red_H^+^/H_sred_H^+^, whose reduction potentials *E*_1_^0′^ and *E*_2_^0^′ depend on pH (as described in eqs 7 and 8 below), and one protonation whose acidity dissociation constant K defines the relative populations of H_red_ and H_red_H^+^. This model can be fitted to the fractions of H_ox_, H_red_, H_red_H^+^, and H_sred_H^+^ as a function of pH (black dashed lines in Figure 2B, see the equations in Supporting Information Section S1.2) by adjusting three parameters: the alkaline limit of *E*_1_^0^′, −359 mV vs SHE, the acidic limit of *E*_2_^0^′, −405 mV, and the value of p*K*, 7.2. All these values are close to those previously measured from the results of spectroelectrochemical experiments.^8^

These data show that a very similar behavior of the Cr HydA1 enzyme is observed under thermodynamic equilibrium in a pH titration as has been observed previously in pseudoequilibrium spectroelectrochemical titrations (ref 8). Moreover, these data, when analyzed with an appropriate thermodynamic model, provide the relevant p*K*_a_ values and redox potentials that can be compared to those derived in kinetic modeling of electrochemistry data, vide infra.

Further analysis of the spectroscopy of Tam HydS will be helpful to fully elucidate the relation between the structures and IR signatures. In the recent pioneering work on Tam HydS, a large hysteresis in the titration of the active site prevented the measurement of thermodynamic parameters.^25^ In combination with the limited number of H-cluster states identified in Tam HydS to date, we chose not to perform the pH titration of Tam HydS in this work, leaving it for future investigations.

### Voltammetry

We examined the catalytic responses of Cr HydA1 and Tam HydS adsorbed onto the surface of a PGE electrode under conditions of direct electron transfer.^33,34^ We recorded cyclic voltammograms at a slow scan rate (so that the obtained current response is always under steady state), rotating and rotated the electrode at a fast rate so that mass transport of the substrate H_2_ toward the electrode is not influential. Recording a single voltammogram gives little information about the catalytic cycle (just like the measurement of a single value of the catalytic rate in a solution assay), but we examined how the steady-state current response depends on pH at a constant H_2_ pressure (1 atm.) and on the concentration of H_2_ (changed by changing the partial H_2_ pressure) at a constant pH (pH 6.5 for Tam HydS and 7.7 for Cr HydA1).

The steady-state catalytic responses of hydrogenases are analyzed here (and in all of our previous work) with a generic rate equation that depends on four parameters: two limiting currents *i*_lim_^ox^ and *i*_lim_^red^ whose values are proportional to the turnover frequency under very oxidizing and very reducing conditions and two “catalytic potentials” *E*_cat_^ox^ and *E*_cat_^red^.^35,37,41^

1 $$i=\frac{i_{\lim}^{ox},\exp^{F\left(E-E_{cat}^{ox}\right)/RT},\exp^{F\left(E-E_{cat}^{red}\right)/RT}-i_{\lim}^{red}}{1+\exp^{F\left(E-E_{cat}^{red}\right)/RT}\left(1+\exp^{F\left(E-E_{cat}^{ox}\right)/RT}\right)}$$

The catalytic potentials are the values of the electrode potential below and above which H_2_ evolution and oxidation, respectively, are observed. They are measured from the positions of the inflection potentials of the catalytic waves, or by fitting a model to the waveshape.^35,37,41^ By definition, irreversible catalysis corresponds to the situation where *E*_cat_^ox^ is significantly greater than *E*_cat_^red^, with a potential range where no catalysis occurs.^29–31,37^

The rate equation must be modified to account for the broadening that results from slow and distributed interfacial electron transfer, as described previously.^35,42^ For this, we assume that electron transfer between the electrode and the active site is direct and that the values of the ET rate constants depend on electrode potential according to the Butler–Volmer equation. This is correct for Cr HydA1, which embeds no accessory clusters, and it is also a good approximation in the case of Tam HydS (despite the probable implication of FeS clusters in mediating long-range electron transfer in this enzyme) on condition that intramolecular electron transfer is fast relative to the turnover of Tam HydS.^35^ Considering slow intramolecular electron transfer would add parameters to a model that already provides a very good fit of the data, so these parameters would necessarily be underdetermined.^35^

The model was then fitted to the voltammograms (see Sections S2.7–S2.9 and ref 31) to measure the catalytic potentials and the high and low potential limiting currents. The latter are related to the equilibrium potential (the Nernst potential of the H^+^/H_2_ couple) by the following equation^37^

2 $$\frac{i_{\lim}^{ox}}{i_{\lim}^{red}}=\exp\left[\frac{2F}{RT}\left(\frac{E_{cat}^{ox}+E_{cat}^{red}}{2}-E_{eq}\right)\right]$$

The steady-state rate equation (eq 1) and the constraint in eq 2 are valid for the steady-state voltammetric response of any two-electron bidirectional catalytic cycle that is “ordered” (meaning that it involves the same series of steps for the forward and backward reactions).^37^ Since the rate equation does not depend on the details of the catalytic cycle such as the order of the catalytic steps, the only conclusion from the observation that the equation fits well the voltammetry is that the catalytic mechanism is ordered. However, important insights come from the detailed interpretation of how the four parameters (catalytic potentials and limiting currents) depend on the experimental parameters, in particular pH and H_2_ concentration, as discussed in ref 40 and illustrated below.

## Modeling

The meaning of the catalytic potentials depends on the order and the rate constants of the steps in the catalytic cycle,^37^ and to interpret the dependence of the catalytic potentials and limiting currents on pH and H_2_ concentration, one has to make explicit assumptions about which rate constants depend on pH and [H_2_].^40^ A kinetic model that would involve all of the steps that have been postulated in the catalytic cycle of FeFe-hydrogenases would depend on too many parameters and would be impractical from the point of view of the kinetic analysis. Like in a recent study of H^+^/H_2_ conversion by a synthetic complex,^39^ the compromise that we found useful was to consider a catalytic cycle model that includes four distinct steps: two redox steps (“E”) and two nonredox steps (“C” for “chemical”), as depicted in Figure 4A.

**Figure 3:**
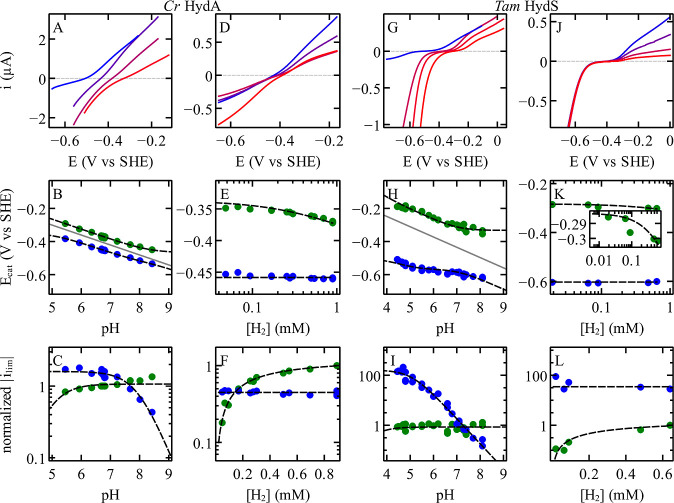
Cyclic voltammograms, catalytic potentials, and limiting currents at different pHs and [H_2_] pressures for Cr HydA1 and Tam HydS. The black dashed lines are the best fits of eqs 11–14, with the parameters shown in Table 1 (deduced from the analysis of voltammetry at different pH values) or the best fits of eqs 23 and 26, with the parameters shown in Table 2 (from the analysis of voltammetry at different [H_2_] concentrations). Panels A and D show selected blank subtracted, averaged, cyclic voltammograms for Cr HydA1 at different pH values (under 1 atm of [H_2_], 30 °C) and [H_2_] pressures (at pH 7.7, 5 °C), respectively. Colors go from blue to red from high to low pH (pH values are 5.4, 6.3, 7.2, and 8.4 in panel A) and from high to low H_2_ partial pressure (0.89, 0.36, 0.18, and 0.04 mM [H_2_] in panel D). Other conditions: scan rate 20 mV/s and electrode rotation rate 3000 rpm. Panels G and J show selected, blank subtracted, averaged cyclic voltammograms for Tam HydS at different pH values (under 1 atm of [H_2_], 40 °C) and [H_2_] pressures (at pH 6.5, 40 °C), respectively. Colors go from blue to red from high to low pH (pH values are 4.8, 5.5, 6, and 8.1 in panel G) and from high to low H_2_ partial pressure (0.64, 0.5, 0.09, and 0.06 mM [H_2_] in panel J). Other conditions: 10 mV/s and 3000 rpm. The catalytic potentials and the normalized limiting currents are plotted as functions of pH (panels B and C for Cr HydA1 and panels H and I for Tam HydS) and as functions of H_2_ concentration (panels E and F for Cr HydA1 and panels K and L for Tam HydS). The current values were normalized by the value of *i*_lim_^ox^ at pH 7 in the plots of i_lim_ against pH and by the value of *i*_lim_^ox^ under 1 atm. of H_2_ in the plots of i_lim_ against [H_2_]. In green *E*_cat_^ox^ and *i*_lim_^ox^ and in blue *E*_cat_^red^ and *i*_lim_^red^. In panels B and H, a solid gray line indicates the Nernst potential of the H^+^/H_2_ couple.

**Figure 4:**
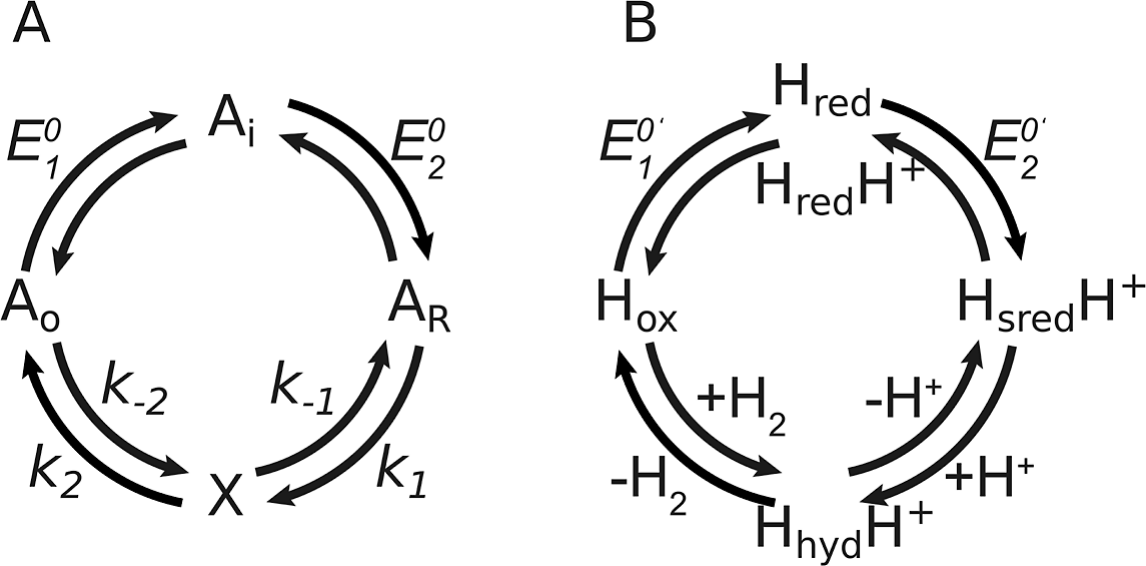
Kinetic schemes discussed in this paper. (A) Generic scheme of the catalytic mechanism EECC,^1^ where A is the active site (in the redox states O, oxidized, I, intermediate, and R, reduced), “X” is a catalytic intermediate, and all steps are reversible. (B) Mechanism adapted to analyze the steady-state kinetics of hydrogenases. The two-electron reduction of H_ox_ to H_sred_H^+^ is coupled to one protonation (hence the pH dependence of *E*_1_^0^′ and *E*_2_^0^′ in eqs 7 and 8), and the first and second chemical steps correspond to protonation (eqs 9 and 10) and H_2_ release/binding (eqs 21 and 22), so that the “X” intermediate corresponds to H_hyd_H^+^ and H_ox_H_2_. Only the former is shown in (B).

The limiting currents and catalytic potentials depend on the values of *E*_1_^0^ and *E*_2_^0^ and on the values of the four rate constants of the two chemical steps, as demonstrated in ref 37

3 $$i_{\lim}^{ox}=2FA\Gamma \frac{k_{-1}k_{-2}}{k_{2}+k_{-1}+k_{-2}}$$

4 $$i_{\lim}^{red}=-2FA\Gamma \frac{k_{1}k_{2}}{k_{1}+k_{2}+k_{-1}}$$

5 $$E_{cat}^{ox}=E_{1}^{0}-\frac{RT}{F}\;\ln\frac{k_{2}+k_{-1}+k_{-2}}{k_{2}+k_{-1}}$$

6 $$E_{cat}^{red}=E_{2}^{0}+\frac{RT}{F}\;\ln\frac{k_{2}+k_{-1}+k_{1}}{k_{2}+k_{-1}}$$

To match the mechanism of model 1 and the Pourbaix diagram in Figure 1C, we assume that the first redox step (the reduction of H_ox_) is coupled to a fast protonation at pH < p*K*, so that its redox potential depends on pH according to

7 $$E_{1}^{0\prime }=E_{1}^{0}+\frac{RT}{F}\;\ln\left[1+\frac{\left[H^{+}\right]}{K}\right]$$

where p*K* is the p*K*_a_ of H_red_ (corresponding to the protonation of the amine bridge) and *E*_1_^0^ is the reduction potential of the H_ox_/H_red_ couple in the alkaline limit, as indicated in Figure 1C. As a consequence, the redox potential of the second redox step is

8 $$E_{2}^{0\prime }=E_{2}^{0}-\frac{RT}{F}\;\ln\left[1+\frac{K}{\left[H^{+}\right]}\right]$$

where *E*_2_^0^ is the reduction potential of the H_red_H^+^/H_sred_H^+^ couple in the acidic limit. These two redox steps correspond to the two-electron, one-proton reduction of H_ox_ into H_sred_H^+^ (Figure 1A).

The reductive catalytic cycle is completed by a series of chemical steps that include the second protonation, and H_2_ binding and release, which we describe in the model by two distinct, bidirectional, nonredox steps “C” (Figure 4A).

A strong assumption is that the H_red_/H_red_H^+^ transition is fast on the turnover time scale and remains at equilibrium during the catalytic cycle, whereas the second protonation is allowed to be slow. This reduces the catalytic cycle to just four steps. This approximation is given justification by the very good agreement between the resulting “EECC” model in Figure 4A and the data, as described below. Making the first protonation also slow (in what would become an “ECECC” scheme) would add parameters to a model that is already very good, so these parameters would necessarily be underdetermined (the situation is the same as that discussed above, regarding our rudimentary description of the electron-transfer kinetics). In our attempts to find the “best” and simplest model, we have also considered an ECEC catalytic cycle, which failed to reproduce the pH dependence (cf. Section S2.10).

### pH Dependence of the Cr HydA1 Voltammetric Response

We first focus on the pH dependence of the catalytic potentials and limiting currents of Cr HydA1. For this, we explicitly include a protonation in the first chemical step as shown in Figure 4B using a realistic description of the (de)­protonation kinetics (we also tested protonation in step 2, but this leads to inconsistencies; cf. Section S2.1). Since the proton is transferred to the H-cluster from the final proton relay (the side chain of a conserved cysteine residue in group A hydrogenases) rather than directly from bulk water or from the buffer, the rate constants of the protonation and deprotonation steps, *k*_1_ and *k*_–1_, respectively, depend on the pH according to

9 $$k_{1}=\frac{k_{1}^{\max}}{1+\frac{K_{relay}}{\left[H^{+}\right]}}$$

10 $$k_{-1}=\frac{k_{-1}^{\max}}{1+\frac{\left[H^{+}\right]}{K_{relay}}}$$

where *K*_relay_ is the acidity constant of the proton relay.^39,43^

Replacing *E*_1_^0^ and *E*_2_^0^ with *E*_1_^0^′ and *E*_2_^0^′ and substituting eqs 9 and 10 into eqs 3–6 give the pH dependence of the two limiting currents and the two catalytic potentials as a function of eight parameters: K, defined in eqs 7 and 8, *E*_2_^0^, defined above, and α, β, *E*_1_^0app^, *K*_1_, *K*_2_, and *K*_3_, all defined below.

11 $$i_{\lim}^{ox}=\frac{\alpha }{1+\frac{\left[H^{+}\right]}{K_{1}}}$$

12 $$i_{\lim}^{red}=\frac{\beta }{1+\frac{K_{3}}{\left[H^{+}\right]}}$$

13 $$E_{cat}^{ox}=E_{1}^{0app}+\frac{RT}{F}\;\ln\left[1+\frac{\left[H^{+}\right]}{K}\right]+\frac{RT}{F}\;\ln\left[\frac{1+\frac{\left[H^{+}\right]}{K_{2}}}{1+\frac{\left[H^{+}\right]}{K_{1}}}\right]$$

14 $$E_{cat}^{red}=E_{2}^{0}-\frac{RT}{F}\;\ln\left[1+\frac{K}{\left[H^{+}\right]}\right]+\frac{RT}{F}\;\ln\left[\frac{1+\frac{\left[H^{+}\right]}{K_{3}}}{1+\frac{\left[H^{+}\right]}{K_{2}}}\right]$$

The parameters α and β in eqs 11 and 12 are pH independent

15 $$\alpha =2FA\Gamma \frac{k_{-2}k_{-1}^{\max}}{k_{2}+k_{-2}+k_{-1}^{\max}}$$

16 $$\beta =-2FA\Gamma \frac{k_{2}k_{1}^{\max}}{k_{2}+k_{1}^{\max}}$$

They are not discussed further because their value defines the magnitude of the voltammetric current, which, in Protein Film Electrochemistry, is proportional to the unknown electroactive coverage, Γ, and thus cannot be interpreted.^33,44^ Four of the six parameters in eqs 11–14 are related to the parameters of the catalytic cycle by the following relations

17 $$E_{1}^{0app}=E_{1}^{0}+\frac{RT}{F}\;\ln\left[\frac{k_{2}+k_{-1}^{\max}}{k_{2}+k_{-2}+k_{-1}^{\max}}\right]$$

18 $$K_{1}=K_{relay}\times \frac{k_{2}+k_{-2}+k_{-1}^{\max}}{k_{2}+k_{-2}}$$

19 $$K_{2}=K_{relay}\times \frac{k_{2}+k_{-1}^{\max}}{k_{2}}$$

20 $$K_{3}=K_{relay}\times \frac{k_{2}+k_{-1}^{\max}}{k_{2}+k_{1}^{\max}}$$

p*K*_1_, p*K*_2_, and p*K*_3_ are “catalytic p*K*_a_’s”,^40^ which depend on the acidity of the proton relay and on kinetic parameters. These apparent p*K*_a_’s defined by eqs 18–20 are not thermodynamic quantities (as explained in ref 40). They do not each correspond to the protonation of a particular intermediate, but they are three because a model that includes three protonation events (here, two at the active site and one at the proton relay) should depend on at most three apparent p*K*_a_’s. Similarly, the value of *E*_1_^0app^ cannot be easily interpreted because it is shifted from *E*_1_^0^, the alkaline limit of *E*_1_^0′^ (cf. eq 17). The other two parameters, *E*_2_^0^ and *K*, are thermodynamic quantities. We used eqs 11–14 to interpret the variations with pH of the catalytic potentials and limiting currents of Cr HydA1 (panels B–F in Figure 3).

Considering eq 11, the observation that *i*_lim_^ox^ is pH independent (green data points in Figure 3C) implies p*K*_1_ < 5. The value of p*K*_3_ ≈ 7.9 could, Figure 4, be directly deduced by fitting eq 12 to the pH dependence of *i*_lim_^red^ (blue in Figure 3C).

The same apparent acidity constants (“*K*_*i*_”) appear in several of eqs 11–14, meaning that the four variations with pH are not independent of one another. Moreover, eqs 18–20 imply that p*K*_2_ is necessarily lower than both p*K*_1_ and p*K*_3_. This gave useful constraints to interpret the pH dependence of the catalytic potentials.

Fitting eq 14 to the pH dependence of *E*_cat_^red^ (green in Figure 3B) using the constraint p*K*_2_ < p*K*_3_ and p*K*_3_ ≈ 7.9 (measured from *i*_lim_^ox^) gave *E*_2_^0^ = −523 mV and p*K* = 8.3 (slightly above the value, 7.2, determined from the data in Figure 2).

Fitting eq 13 to the pH dependence of *E*_cat_^ox^ (blue in Figure 3B) with the constraint p*K*_2_ < p*K*_1_ (compare eqs 18 and 19), with p*K* = 8.3 (from the above fit of the pH dependence of *E*_cat_^red^), and p*K*_1_ < 5 (from the pH dependence of *i*_lim_^ox^) returned *E*_1_^0app^ (which is lower than *E*_1_^0^′, cf. eq 17). Although it is difficult to estimate an error on the value of p*K*, we observed that a value of p*K* = 7.8 (instead of the best value of 8.3) also gives a fit that is acceptable, with a value of *E*_1_^0app^ that is 30 mV more positive than its best value.

These Cr HydA1 parameters are collected on the first row of Table 1. The values of some of the parameters can only be specified as an upper limit (e.g., p*K*_1_ < 5) because any value lower than that indicated gives an equally good fit of the data.

**Table 1:** Parameters Obtained by Fitting Eqs 11–14 to the Data Shown in Panels B, C, H and I of Figure 3 to Interpret the pH Dependence of the Catalytic Potentials and Limiting Currents^a^

	*E*_1_^0app^ (mV)	*E*_2_^0^ (mV)	p*K*	p*K*_1_	p*K*_2_	p*K*_3_
Cr HydA1	–466	–523	8.3	<5	<5	7.9
Tam HydS	–332	–568	7.1	<3.5	<4	5.1

a *E*_1_^0app^ is distinct from *E*_1_^0^ (cf. eq 17).

### Dependence on H_2_ Concentration of the Cr HydA1 Voltammetric Response

To explain the dependence of the catalytic potentials and limiting currents on [H_2_] at a constant pH, we included the binding and release of H_2_ in step 2, Figure 4B, assuming for simplicity that the release of H_2_ is unimolecular

21 $$k_{2}=cst$$

and H_2_ binding is bimolecular

22 $$k_{-2}=k_{-2}^{\prime }\times \left[H_{2}\right]$$

Considering more complex H_2_-binding kinetics (as described in Section S2.3) is not useful: it accounts for the slight inhibition by H_2_ of H_2_ evolution, but it does not change the conclusions of this work while adding to the model parameters that cannot be determined.

Substituting eqs 21 and 22 in eqs 3–6 gave

23 $$i_{\lim}^{ox}=2FA\Gamma \frac{k_{-1}}{1+\frac{K_{M}}{\left[H_{2}\right]}}$$

24 $$i_{\lim}^{red}=-2FA\Gamma \frac{k_{1}k_{2}}{k_{1}+k_{2}+k_{-1}}$$

25 $$E_{cat}^{ox}=E_{1}^{0\prime }-\frac{RT}{F}\;\ln\left[1+\frac{\left[H_{2}\right]}{K_{M}}\right]$$

26 $$E_{cat}^{red}=E_{2}^{0app}=E_{2}^{0\prime }+\frac{RT}{F}\;\ln\frac{k_{2}+k_{-1}+k_{1}}{k_{2}+k_{-1}}$$

with

27 $$K_{M}=\frac{k_{2}+k_{-1}}{k_{-2}^{\prime }}$$

We fitted eqs 23–27 to interpret the dependence of the limiting currents and catalytic potentials recorded at a constant pH on the H_2_ concentration.

The reductive limiting current is nearly independent of H_2_ (blue in Figure 3F), consistent with eq 24. The change in *i*_lim_^ox^ against [H_2_] (green in Figure 3F and eq 23) returned a value of *K*_M_ = 0.3 mM that is consistent with previous measurements of ours.^45^

Equation 26 is consistent with the observation that *E*_cat_^red^ is independent of H_2_ pressure. Fitting a horizontal line to *E*_cat_^red^ (blue in Figure 3E) gave the value of *E*_2_^0app^ reported in Table 2, which is offset from the value of *E*_2_^0^′ (cf. eq 26).

**Table 2:** Parameters Obtained by Fitting Eqs 23–27 to the Data Shown in Panels E, F, K, and L of Figure 3 to Interpret the Dependence on [H_2_] of the Catalytic Potentials and Limiting Currents at pH = 7.7 (Cr HydA1) and 6.5 (Tam HydS)^a^

	*E*_1_^0^′ (mV)	*E*_2_^0app^ (mV)	*K*_M_ (mM)
*Cr* HydA1	–337	–458	0.3
*Tam* HydS	–283	–603	0.6

a The corresponding Cr HydA1 data (Figure 3E,F) were recorded at 5 °C, where the *K*_M_ value is lower,^45^ and the variations of *E*_cat_^ox^ and *i*_lim_^ox^ against H_2_ are more clearly seen. Here, the value of parameter *E*_2_^0app^ is offset from *E*_2_^0^′ (cf. eq 26) and thus cannot be interpreted.

Equation 25 accounts for the observed decrease in *E*_cat_^ox^ as the concentration of H_2_ increases above the value of *K*_M_ (green in Figure 3E). The effect is small because the value of *K*_M_ is high (even at the low temperature, 5 °C, that we used in this series of experiments), and it is not possible to record data at H_2_ concentrations well above *K*_M_. Yet, the trends are clear and fully consistent with eqs 23–27.

### Analysis of the Tam Data

We used the same kinetic model, the same assumptions, and the same approach to analyze the “irreversible” voltammograms obtained with the enzyme from Tam HydS (panels G–L in Figure 3). The only difference was that, as explained before,^31^ the equilibrium potential was measured using a platinized electrode, and its value was used to constrain the fit (using eq 2) to obtain well-defined values of the catalytic potentials; this was not needed for the Cr HydA1 data because the Cr HydA1 catalytic current sharply crosses the potential axis at *E* = *E*_eq_.

The variations with pH and [H_2_] of the limiting currents (panels I and L in Figure 3) are similar to those observed with Cr HydA1 (panels C and F).

The value of *K*_M_ is larger than that of Cr HydA1 (cf. Table 2), but this is explained by the Tam HydS experiments being carried out at 40 °C, compared to 5 °C for the dependence on [H_2_] of the Cr HydA1 data (a high temperature was required because the activity of Tam HydS and the catalytic currents are small), and the value of *K*_M_ increases with temperature.^45^ We confirmed this *K*_M_ value at 40 °C, measured from the CVs in Figure 3, by the chronoamperometry experiments shown in Supporting Information Section S2.6. The large value of *K*_M_ makes the dependence of *E*_cat_^ox^ on [H_2_] in Tam HydS flatter than in Cr HydA1, but the increase in *E*_cat_^ox^ at low H_2_ concentrations is clearly observed (inset in Figure 3K), consistent with eq 25. This cannot be explained by assuming H_2_ binding in step 1 (cf. Supporting Information Section S2.2).

Also, the value of p*K*_3_ is significantly lower in Tam HydS than in Cr HydA1 (cf. Table 1) which can be seen from the saturation of the H_2_ evolution current below a lower value of the pH, compare panels C and I in Figure 3. For Tam HydS, the value of p*K*_3_ = 5.1 measured from the pH dependence of *i*_lim_^red^ (blue in Figure 3I) is also clearly seen as an inflection in the pH dependence of *E*_cat_^red^ (green in Figure 3H). The larger difference between p*K* and p*K*_3_ in Tam HydS compared to Cr HydA1 (still with values of p*K*_1_ and p*K*_2_ outside the experimental pH range) explains the nonlinear pH dependence of the catalytic potentials seen in Figure 3H.

The *K*_M_ value measured here for Tam HydS is close to that measured for other FeFe hydrogenases, despite the putative implication of Tam HydS in sensing rather than catalysis. That the Michaelis constants of FeFe-hydrogenases are apparently all similar contrasts with the situation observed with other series of homologous enzymes: the *K*_M_ values of CO-dehydrogenases, for example, range over orders of magnitude.^46^ However, a Michaelis constant is a convoluted kinetic parameter (eq 27) whose meaning is not straightforward.

## Discussion

Protein film voltammetry gives the dependence of activity on electrochemical driving force, from which “catalytic potentials” can be measured.^37,41^ In the case of H^+^/H_2_ conversion by hydrogenases, these are the values of the electrode potentials below and above which H_2_ evolution and oxidation, respectively, are observed. How these values depend on substrate concentration can be interpreted to learn about the sequence of events in the catalytic cycle,^33,47,48^ but in the particular case of hydrogenases, that the protonation steps may be slow on the time scale of turnover significantly complicates their interpretation: the catalytic potentials depart from the equilibrium reduction potentials measured in redox titrations.

The catalytic potentials can be measured without making any assumption about the catalytic cycle; however, their values depart from the true (equilibrium) reduction potentials of the active site, and their meaning depends on the details of the catalytic cycle. Similarly, we have shown before that if protonation is slow on the time scale of turnover, the dependence of the catalytic potentials and limiting currents on pH defines apparent p*K*_a_’s, which we called “catalytic p*K*_a_’s”. These p*K*_a_’s are kinetic parameters whose interpretation is model dependent.^40^ That a catalytic system under steady-state turnover conditions defines apparent parameters (apparent potentials, acidity, or dissociation constants) is not unexpected: it is textbook knowledge that a Michaelis–Menten constant is an apparent dissociation constant that depends on all steps in the catalytic cycle rather than a true thermodynamic parameter.

Here, we interpreted and compared the variations with pH and H_2_ pressure of the catalytic potentials and limiting currents of the “reversible” hydrogenases, Cr HydA1, and the “irreversible” one, Tam HydS, using a kinetic model of catalysis based on the current knowledge of the catalytic cycle (model 1 in Figure 1D). According to this model, the most oxidized H_ox_ state is reduced in two one-electron-transfer steps, one of which is coupled to protonation, and the resulting H_sred_H^+^ species undergoes protonation before it releases H_2_. Model 2 could not be used to analyze the voltammetric data because the transition from H_red_’ to the low pH inactive branch (H_red_ and H_sred_ in Figure 1E) and from H_ox_H to the presumably less active H_ox_ state at high pH should induce a decrease in proton reduction activity as the pH is lowered, and a decrease in H_2_ oxidation activity as the pH is increased, neither of which are observed under conditions of direct electron transfer (panels C and I in Figure 3). The pH-independent, slow formation of reduced inactive states has been observed in FeFe-hydrogenases, under conditions that are more reducing than those used in this work, but they have not been associated with specific spectroscopic signatures.^49,50^

Interpreting the kinetics of the catalytic cycle in the context of model 1 implies the pH dependences of the reduction potentials of the two ET steps given by eqs 7 and 8 and the dependence on pH and H_2_ concentration of the two nonredox steps given by eqs 9, 10, 21, and 22, respectively. Substitution in eqs 3–6 gave relations [Disp-formula eq11]–[Disp-formula eq14] and 23–26, which successfully predict the variations in limiting currents and catalytic potentials as a function of pH at a constant H_2_ pressure and as a function of H_2_ at a constant pH.

In contrast, considering either protonation in step 2 or H_2_ release in step 1 does not give a consistent description of the data. With this hypothesis, eqs 11–14 remain valid, but the meaning of the catalytic pKs and the constraints differ (Supporting Information Section S2.1). Fitting this model to the pH variations of *E*_cat_ and *i*_lim_ of Cr HydA1 and Tam HydS returns p*K* < 5 and p*K* < 4, respectively. This is not consistent with the value of p*K* = 7.2 measured in Cr HydA1.^8^ A low value of p*K* is expected if we consider the catalytic cycle of model 2 (without the inactive states, that is, H_ox_H → unprotonated H_red_′ → H_hyd_ → H_ox_H, solid arrows in Figure 1E); this mechanistic hypothesis may explain the observed dependence on pH of the Cr HydA1 data on condition that H_2_ release is in step 1, and protonation in step 2, but then, this model predicts the wrong variation of *E*_cat_^ox^ with [H_2_] (Supporting Information Section S2.2). Therefore, we were not able to reconcile model 2 with the Cr HydA1 data.

For the same reasons, analysis of the Tam HydS voltammetry gives a p*K* value for the half-reduced state close to 7. This is not consistent with the spectroscopic investigation of Tm HydS, which did not detect any protonated state of the H-cluster.^24^ This will have to be investigated further by examining how the reduction potentials of the active sites of Tm and Tam HydS depend on pH, which is probably the easiest way to detect the coupling between protonation and reduction, which is assumed in our kinetic model and which necessarily occurs in the catalytic cycle of hydrogenases.

Regarding the analysis of the Cr HydA1 data, the parameters that we deduced by fitting eqs 11–14 and 23–26 (Tables 1 and 2, respectively) are consistent with the results of previous investigations: the p*K*_a_ value of the one-electron reduced state (p*K* ≈ 8.3) is close enough to that measured in redox titrations (7.2 in ref 8) and pH titrations (p*K* around 7.5 in Figure 5C of ref 9 and 7.2 from the data in Figure 2); the value of *K*_M_ also matches to previous chronoamperometric measurements.^45^ It is not possible to deduce the alkaline limit *E*_1_^0^ from the analysis of the pH dependence of the catalytic potentials because it is shifted from the value of the parameter that we can measure, *E*_1_^0app^, cf. eq 17. However, the analysis of the dependence on H_2_ concentration at a constant pH gives the value of *E*_1_^0^′ at pH = 7.7, −337 mV, which matches the value of −362 mV at pH 8 in ref 8 (this value is confirmed from the analysis of the data in Figure 2). The acidic limit of *E*_2_^0^′ obtained from the analysis of the voltammetry at different pH values (*E*_2_^0^ = −523 mV at 30 °C, Table 1) is lower than the value of −417 mV at 15 °C measured in ref 8 and −405 mV at room temperature from the equilibrium FTIR data in Figure 2. However, the reduction potentials are temperature dependent, and the analysis of the pH dependence of the voltammetry at 5 °C gives *E*_2_^0^ = −457 mV (Supporting Information Section S2.5), which we consider close enough to the results of the spectroscopic titrations.

Regarding Tam HydS, there are no available results of equilibrium titrations that we could compare to the values of *E*_2_^0^ in Table 1 and *E*_1_^0^′ in Table 2 since the titration in Figure S8 of ref 25 shows a very strong hysteresis that attests to a strong departure from equilibrium. However, the titration of Tm HydS in ref 24 is consistent with the larger thermodynamic stability of H_red_/H_red_H^+^ that we detect in Tam HydS: the result of the titration gave *E*_1_^0^′ = −300 and *E*_2_^0^′ = −570 mV in Tm HydS at pH 8 ^24^, while the analysis of the voltammetry of Tam HydS returns *E*_1_^0^′ = −283 at pH 6.5 (Table 2) and *E*_2_^0^ (the acidic limit of *E*_2_^0^′) = −568 mV (Table 1).

We assumed that the same mechanism applies in Cr HydA1 and Tam HydS (Figure 5), but their catalytic waveshapes are different, and so are the parameters that we measured from the analyses of the variations with pH and H_2_ of their catalytic potentials and limiting currents. These differences between Cr HydA1 and Tam HydS are all consistent with an increased irreversibility of the catalytic response in Tam HydS compared to Cr HydA1 (i.e., a larger separation between the oxidative and reductive catalytic potentials).

**Figure 5:**
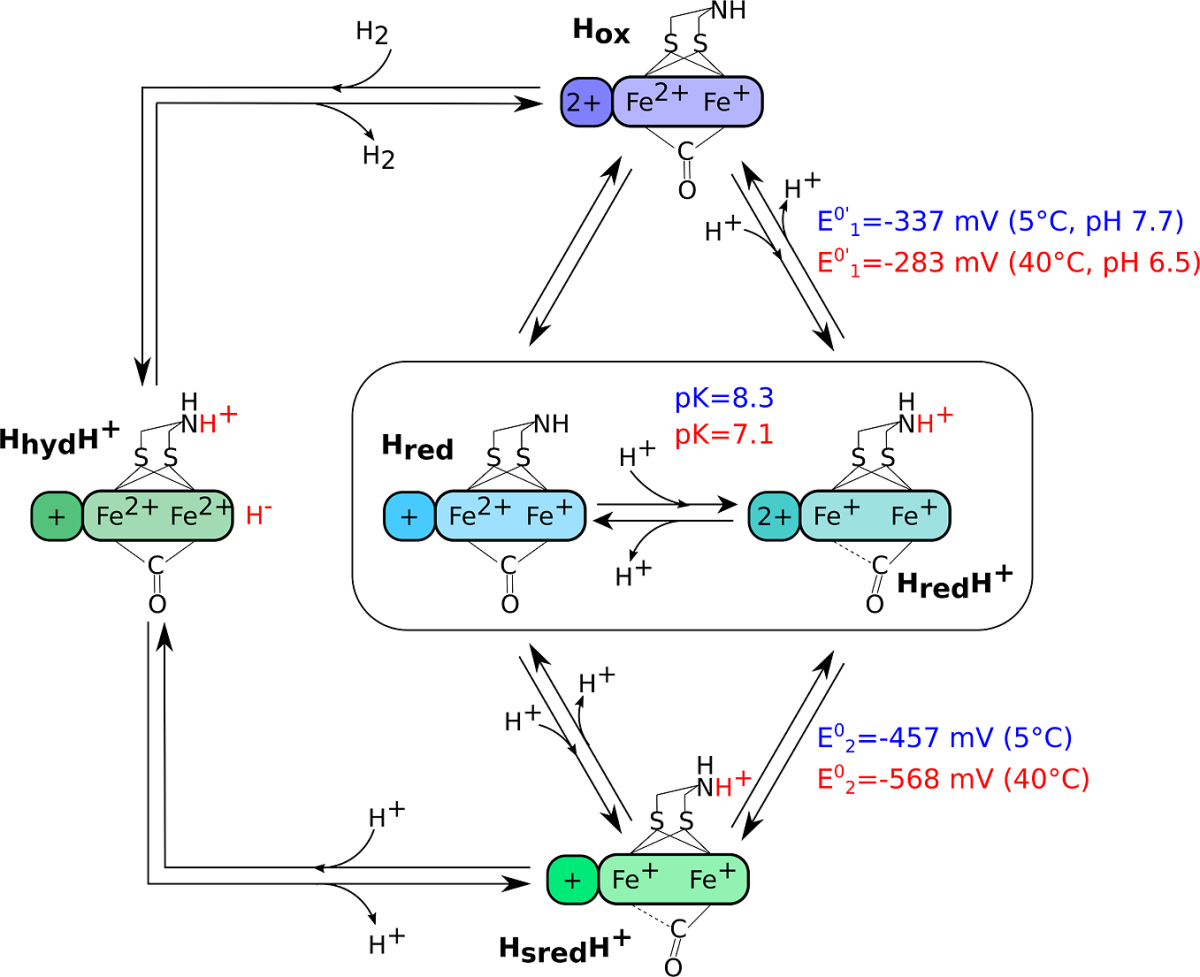
Proposed catalytic cycle for FeFe-hydrogenases. The intermediate states of the catalytic cycle are colored as in Figure 1. The values of *E*_1_^0^′, *E*_2_^0^, and p*K* obtained by fitting the voltammetry of Cr HydA1 and Tam HydS at different pH values and H_2_ partial pressures are marked on the right side of the figure in blue and red, respectively. The values of *E*_1_^0^′ were obtained by the analysis at different H_2_ pressures, at a specific pH, reported in the figure. *E*_2_^0^ is measured from the analysis at different pHs, and it is the acidic limit of *E*_2_^0^′, independent of pH when pH < p*K*. In the kinetic model, the H_red_/H_red_H^+^ transition (inside the rectangle) is assumed to remain in equilibrium.

Our data show that the half-reduced catalytic intermediate (considering both H_red_ and H_red_H^+^) is stable over a range of potential that is about 165 mV larger in Tam HydS than in Cr HydA1, which results from both *E*_1_^0^ being more positive in Tam HydS than in Cr HydA1 and *E*_2_^0^ being more negative (Tables 2 and 1, respectively). This thermodynamic stability of the half-reduced state contributes to making the catalytic response of Tam HydS very irreversible.

In contrast, the unstable nature of the half-reduced active site is a key feature of bidirectional reversible catalysts.^32,37^ The difference *E*_1_^0^′ – *E*_2_^0^′ is indeed less positive in Cr HydA1 than in Tam HydS, and even negative in the case of two recently described bidirectional reversible synthetic catalysts. The +1 redox state of the [Pt­(depe)_2_]­[PF_6_]_2_ (depe = 1,2-bis­(diethylphosphino)­ethane) complex that converts CO_2_ and formate in acetonitrile is inherently unstable, and the reduction of [Pt­(depe)_2_]^2+^ is therefore a cooperative two-electron process.^51,52^ The recent investigation of the [Ni­(P_2_^Cy^N_2_^Arg^)_2_]^6+^ Dubois complex that reversibly converts H^+^ and H_2_ ^30,53^ also showed that the reduction potential of the Ni^II^/Ni^I^H^+^ couple is much lower than that of the more reduced Ni^III^H^–^/Ni^II^H^–^ couple, which makes the half-reduced tautomers Ni^I^H^+^ and Ni^III^H^–^ unstable.^39^ In both cases, therefore, potential inversion^54^ destabilizes the half-reduced form of the active site and thus decreases *E*_cat_^ox^ – *E*_cat_^red^ and contributes to make the response reversible.

Another difference between the two hydrogenases can be seen as a lower value of p*K*_3_ in Tam HydS compared to Cr HydA1 (Table 1), which contributes to a decrease in *E*_cat_^red^ (cf. eq 14). Considering the definition of *K*_3_ in eq 20, the lower value of p*K*_3_ can be interpreted in two different manners. One explanation is that the proton-transfer relay has lower p*K*_a_ (the acidity constant is “*K*_relay_” in eqs 18–20), which is consistent with the recent finding that the proton donor to the active site is the side chain of a glutamate residue in Tam HydS,^27^ compared to a cysteine residue in Cr HydA1 and other prototypical hydrogenases.^3^ The other explanation applies if the rate constant of H_2_ binding at the active site is smaller than the (de)­protonation rate constants. In that case, *K*_3_ equates *k*_–1_^max^/*k*_1_^max^ *K*_relay_, which is the acidity constant of the doubly reduced catalytic intermediate (cf. Section S2.4), and our analysis suggests that this intermediate is harder to protonate in Tam HydS than in Cr HydA1. This protonation, which is required to close the catalytic cycle, has so far proven challenging to firmly identify in spectroscopic investigations.^16,19^

Overall, our results point to various functional differences between Cr HydA1 and Tam HydS, which all contribute to make the catalytic response of the former more reversible: the lower stability of the half-reduced state (smaller difference between *E*_1_^0^′ and *E*_2_^0^′, which has not been rationalized yet in terms of active site proteic environment^28^) and the easier protonation of the relay or of the two-electron reduced state (larger p*K*_3_).

We conclude on the consideration, which is clear from eq 25, that the role played by the stabilization of the enzyme–substrate complex (H_hyd_H^+^) is decreasing the oxidative catalytic potential and therefore increasing the reversibility of the catalytic signal. An experimental comparison between Cr HydA1 and Tam HydS in this respect is difficult because the Michaelis constants are difficult to measure accurately and impossible to measure and compare at the same temperature. However, eq 25 is another illustration of the idea that flat energy landscapes are not required to obtain a reversible catalytic response:^37,39^ that the catalytic cycle that includes high or low energy intermediates may favor reversibility, sometimes at the expense of turnover frequency.

## Methods

The samples of Cr HydA1 and Tam HydS were produced by heterologous expression in followed by artificial maturation with the [2Fe]^adt^ cofactor as described in references with minor modifications (for details, see the Supporting Information).^25,55,56^

The FTIR measurements were performed in a home-built cell in which a sample of 12 μL of 0.3 mM Cr HydA1 in mixed buffer (20 mM each of acetate, MES, HEPES, Tris, glycine, CAPS) prepared under a 2% H2/98% N2 atm in an anaerobic chamber (Coy) was closed between two CaF_2_ windows separated by a 50 μm Teflon spacer. The sample so composed was sealed into the home-built cell with two rubber rings separating the windows from the rest of the housing. The data were recorded in a standard transmission IR spectrometer (a Bruker Vertex v80) equipped with a liquid nitrogen cooled mercury–cadmium-telluride (MCT) detector.

All the electrochemistry experiments were carried out in a Jacomex glovebox filled with nitrogen. The three-electrode electrochemical setup was described in ref 57. The electrochemical cell solution was continuously flushed with either pure H_2_ or a mixture of H_2_ and Ar adjusted to the desired composition using mass flow controllers (SLA5850S from BROOKS Instruments). The resulting H_2_ concentration was calculated from the H_2_ partial pressure assuming a solubility of 0.89 mM/atm at 5 °C and 0.64 mM/atm at 40 °C.^58,59^

The films of Cr HydA1 on PGE electrodes were prepared by letting adsorb 0.5 μL of enzyme solution (5–20 μM) for about 3 min, after having polished the electrode surface (∼0.1 cm^2^) with 1 μm aqueous alumina slurry. The films of Tam HydS were prepared by polishing the electrode surface with 1 μm aqueous alumina slurry and P1200 sandpaper. After sonication, 1 μL of enzyme solution (5–10 μM) was painted on the electrode surface together with 1 μL of polymyxin B sulfate (2–20 mg/mL) and let dry for 5–10 min. The platinized electrode to measure the H^+^/H_2_ equilibrium potential during the Tam HydS experiments was prepared according to the protocol in ref 60.

All of the electrochemical experiments were performed in a chloride-free mixed buffer: MES, CHES, HEPES, TAPS, Na acetate (all 5 mM), and Na_2_SO_4_ (0.1 M).

All potentials were measured with respect to a saturated calomel electrode and then corrected for the SHE by using *E*_SHE_ = *E*_SCE_ + 241 mV.

We accounted for film loss during the series of voltammograms by always recording one CV at pH 7 or at 1 mM [H_2_] between every experiment at a different pH or H_2_ pressure, respectively, and normalizing the limiting currents (as, e.g., in ref 61). Only in the series of experiments with Tam HydS as a function of pH was this not necessary because the films were very stable. The capacitive current was removed by subtracting a blank recorded with no enzyme, and the forward and backward sweeps averaged.

The data were analyzed using the Qsoas software, available at www.qsoas.org.^62^

## Supplementary Material



## Notes

Additional experimental details, materials, and methods regarding the experiment in Figure 2; additional kinetic models and their discussions; fits of *E*_cat_ and *i*_lim_ as a function of pH at 5 °C for Cr HydA1; Tam HydS *K*_M_ measurement; analysis of the cyclic voltammograms of Cr HydA1 and Tam HydS recorded under various conditions; and preparation of [2Fe]­ADT-activated Tam HydS (PDF)
