# Supplementary material for: Kinetic Modeling of the Reversible or Irreversible Electrochemical Responses of FeFe-Hydrogenases
Source: J Am Chem Soc. 2024 Jan 2;146(2):1455–66. doi: 10.1021/jacs.3c10693 (PMC12326359; doi:10.1021/jacs.3c10693)
Supplement: Supplementary file 1 [file ja3c10693_si_001.pdf]

Kinetic modeling of the reversible or irreversible electrochemical responses of FeFe-hydrogenases

Supplementary information

Andrea Fasano[a], Carole Baffert[a], Conrad Schumann[b], Gustav Berggren[b], James A. Birrell[c], Vincent Fourmond[a], Christophe Léger[a*]

a. Laboratoire de Bioénergétique et Ingénierie des Protéines. CNRS, Aix Marseille Université, UMR 7281. Marseille. France.

b. Molecular Biomimetics, Department of Chemistry, Ångström Laboratory, Uppsala University, 75120 Uppsala, Sweden

c. School of Life Sciences, University of Essex, Wivenhoe Park, Colchester, CO4 3SQ, UK

Section S1. FTIR titrations **1**
Section S1.1 Analysis of the FTIR data 1
Section S1.2 Modeling 2
Section S2. Electrochemistry **3**
Section S2.1 (De)protonation in the 2nd chemical step (k_2, k_{-2}) 3
Section S2.2 H_2 binding/release in the 1st chemical step (k_1 and k_{-1}) 5
Section S2.3 The kinetics of H_2 binding and release 7
Section S2.4 Relation between the acidity constant for the second protonation and K_3 10
Section S2.5 Modeling of Ecat and i_{lim} as a function of pH at 5°C for Cr HydA1 12
Section S2.6 Tam HydS KM measurement 13
Section S2.7 Modeling of the cyclic voltammograms of Cr HydA1 recorded at different pH values 13
Section S2.8 Modeling of the voltammograms recorded with Cr HydA1 at different concentrations of H_2. 14
Section S2.9 Modeling of the voltammograms recorded with Tam HydS at different concentrations of H_2 14
S2.10 Modeling the (de)protonation events for an ECEC kinetic scheme 18
Section S3. Preparation of [2Fe]ADT- activated TamHydS **20**
References **21**

Section S1. FTIR titrations

Section S1.1 Analysis of the FTIR data

The fraction of each species shown in figure 2B of the main text was computed assuming that the total sample of enzyme exists in any (or in a mixture) of the following four spectroscopic states: H_{ox}, H_{red}, $H_{red}H^+$ and $H_{sred}H^+$. The sum of the absorbances at the specific wavenumber of each state (1939 cm^{-1}, 1933 cm^{-1}, 1891 cm^{-1} and 1881 cm^{-1}, respectively) is directly correlated with the total amount of enzyme in the sample, from which

the fraction of each state was calculated according to the absorbance of the specific bands. No correction for the extinction coefficient was performed since the sum of the absorbances at the wavenumber of each four species is constant at every pH (figure S1.1), suggesting that these four species have very similar extinction coefficients.

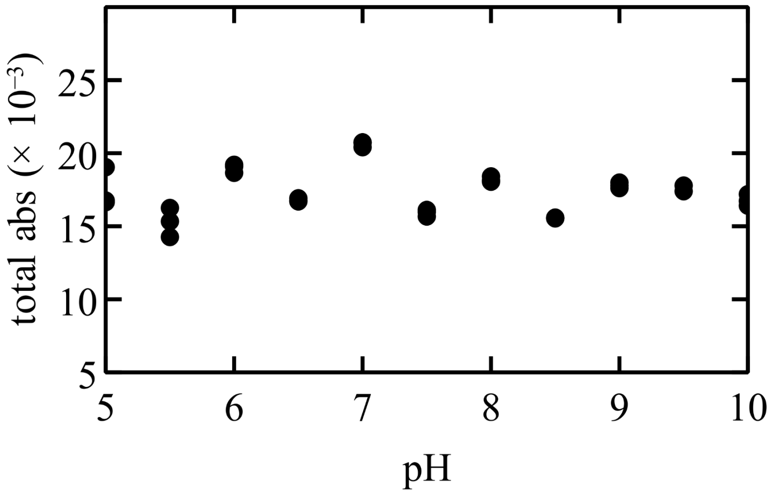

Figure S1.1: sum of the absorbances of the four spectroscopic species H_{ox} (1939 cm^{-1}), H_{red} (1933 cm^{-1}), $H_{red}H^+$ (1891 cm^{-1}) and $H_{sred}H^+$ (1881 cm^{-1}) at every pH.

Section S1.2 Modeling

We have used a simplified version of the six states model in figure 3 of ref [1]. Two of those states are predicted to exist only at very extreme conditions, and have not actually been observed ($H_{ox}H^+$ probably only forms at very low pHs and high potentials and H_{sred} at very high pH and low potentials), which we do not reach in our titration in figure 2 of the main text. The model considers therefore only the redox transition between H_{ox} and H_{red} (reduction potential $E^{0'}_1$); the protonation equilibrium between H_{red} and $H_{red}H^+$, defined by the acidity dissociation constant K; the second redox transition $H_{red}H^+/H_{sred}H^+$ (reduction potential $E^{0'}_2$).

The ratio H_{ox}/H_{red} and $H_{red}H^+/H_{sred}H^+$ is defined by the Nernst equation as:

$$\frac{[\mathrm{H_{ox}}]}{[\mathrm{H_{red}}]} = \exp\left(\frac{F}{RT}(\mathrm{E} - \mathrm{E}_0^1)\right) = e_1$$

$$\frac{[\mathrm{H_{red}H^+}]}{[\mathrm{H_{sred}H^+}]} = \exp\left(\frac{F}{RT}(\mathrm{E} - \mathrm{E}_0^2)\right) = e_2$$

The definition of the acidity constant K implies that

$$[\mathrm{H_{red}}] = \frac{[\mathrm{H_{red}H^+}] \times \mathrm{K}}{[\mathrm{H^+}]}$$

We note C_0 the total concentration of enzyme:

$$[\mathrm{H_{ox}}] + [\mathrm{H_{red}}] + [\mathrm{H_{red}H^+}] + [\mathrm{H_{sred}H^+}] = \mathrm{C_0}$$

Substituting in the last equation each of the terms with the previous definitions and solving it per each species gives the relative populations of each of the four states. A precise description of the entire procedure is in ref [2].

$$[\mathrm{H_{ox}}] = \frac{\mathrm{C_0}}{1 + \frac{1}{\mathrm{e_1}} + \frac{[\mathrm{H^+}]}{\mathrm{Ke_1}} + \frac{[\mathrm{H^+}]}{\mathrm{Ke_1e_2}}}$$

$$[\mathrm{H_{red}}] = \frac{\mathrm{C_0}}{1 + \mathrm{e_1} + \frac{[\mathrm{H^+}]}{\mathrm{K}} + \frac{[\mathrm{H^+}]}{\mathrm{Ke_2}}}$$

$$[\mathrm{H_{red}H^+}] = \frac{\mathrm{C_0}}{1 + \frac{1}{\mathrm{e_2}} + \frac{\mathrm{K}}{[\mathrm{H^+}]} + \frac{\mathrm{Ke_1}}{[\mathrm{H^+}]}}$$

$$[\mathrm{H_{sred}H^+}] = \frac{\mathrm{C_0}}{1 + \mathrm{e_2} + \frac{\mathrm{Ke_2}}{[\mathrm{H^+}]} + \frac{\mathrm{Ke_1e_2}}{[\mathrm{H^+}]}}$$

Section S2. Electrochemistry

Section S2.1 (De)protonation in the 2nd chemical step (k_2, k_{-2})

We assume that the proton is transferred from a proton relay, in the second chemical step

$$k_{+2} = \frac{k_{+2}^{\mathrm{max}}}{1 + \frac{K_{\mathrm{relay}}}{[\mathrm{H^+}]}}$$

$$k_{-2} = \frac{k_{-2}^{\mathrm{max}}}{1 + \frac{[H^+]}{K_{\mathrm{relay}}}}$$

The equations giving E_{cat} and I_{lim} as a function of pH are like eqs 11-14 in the main text. This implies that fitting the experimental data (figure 3 panel B, C, H and I) assuming a protonation in the first or second chemical step returns equally good fits. The difference is in the definition of the apparent potentials and pKs, and therefore on the constraints on the values of the parameters that result.

$$\mathrm{E_1^{0app}} = \mathrm{E_1^0} + \frac{RT}{F} \ln \left[\frac{k_{-1}}{k'_{-2} + k_{-1}} \right]$$

$$\mathrm{E_2^{0app}} = \mathrm{E_2^0} + \frac{RT}{F} \ln \left[\frac{k_1 + k_{-1}}{k_{-1}} \right]$$

$$K_1 = K_{relay} \times \frac{k'_{-2} + k_{-1}}{k'_2 + k_{-1}}$$

$$K_2 = K_{relay} \times \frac{k_{-1}}{k'_2 + k_{-1}}$$

$$K_3 = K_{relay} \times \frac{k_1 + k_{-1}}{k'_2 + k_1 + k_{-1}}$$

$$\alpha = \frac{k'_{-2} k_{-1}}{k'_{-2} + k_{-1}}$$

$$\beta = \frac{k'_2 k_1}{k'_{-2} + k_1 + k_{-1}}$$

(Note that these parameters $E^0{}_1{}^{app}$, $E^0{}_2{}^{app}$ and the apparent acidity constants K_1, K_2, and K_3 are not the same as in eqs 17-20 and 26 in the main text.) The above definitions imply that $pK_1<pK_2$ and $pK_3<pK_2$, and the pH dependence of the catalytic potentials can only be fitted with $pK<pK_1<pK_2$ ($E_{cat}{}^{ox}$) and $pK<pK_3<pK_2$ ($E_{cat}{}^{red}$). The values of the resulting best parameters are listed in table S2.1. As mentioned in the main text, for Cr HydA1 a pK<5 (Table S2.1) is not consistent with spectroelectrochemical pH titration[1] and the H_2 pressure and pH titrations performed without electrochemical control of the potential (Figure 2 in the main text and ref [3]), which both return pK ≈ 7.2. We also consider a pK < 4 for Tam HydS inconsistent with the fact that the enzyme is active over a large range of pHs[4] and that modeling H_2 binding in the first chemical step does not fit the experimental data (section S2.2).

	$E^{0app}{}_1$ (mV)	$E^{0app}{}_2$ (mV)	pK	pK_1	pK_2	pK_3
Cr HydA1	-466	-321	<5	<5	8.3	7.9
Tam HydS	-332	-377	<4	<3.5	7.1	5.1

Table S2.1 Values of the apparent potentials and pK when assuming that the pronation occurs in the second chemical step.

Section S2.2 H_2 binding/release in the 1st chemical step (k_1 and k_{-1})

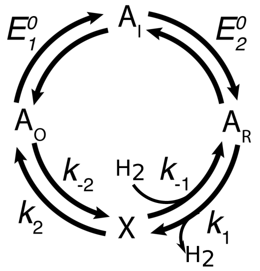

Figure S2.1: general scheme of the EECC model, in which H_2 binding/release occurs in the first chemical step. The rate constants have a negative subscript in the direction of H_2 oxidation and a positive subscript in the direction of H_2 evolution: k_{-1} is the pseudo-1st order rate constant of H_2 binding and k_1 the 1st order rate constant of H_2 release.

In this section, we discuss the equations for E_{cat} and I_{lim} as a function of $[H_2]$, when it is assumed that H_2 binding and release occur in chemical step 1 (figure S2.1).

$$k_1 = \text{cst}$$

$$k_{-1} = k'_{-1} \times [H_2]$$

The changes in E_{cat} and I_{lim} as a function of $[H_2]$ are

$$\mathrm{i}_{\mathrm{lim}}^{\mathrm{ox}} = 2FA\Gamma \frac{k_{-2}}{1 + \frac{K_\mathrm{M}}{[H_2]}}$$

$$\mathrm{i}_{\mathrm{lim}}^{\mathrm{red}} = -2FA\Gamma \frac{\frac{k_1 k_2}{k_1 + k_2}}{1 + \frac{[H_2]}{K_i}}$$

$$\mathrm{E}_{\mathrm{cat}}^{\mathrm{ox}} = \mathrm{E}_1^{0\mathrm{app}} - \frac{RT}{F} \ln \left[\frac{1 + \frac{[H_2]}{K_\mathrm{M}}}{1 + \frac{[H_2]}{K_b}} \right]$$

$$\mathrm{E}_{\mathrm{cat}}^{\mathrm{red}} = \mathrm{E}_2^{0\mathrm{app}} + \frac{RT}{F} \ln \left[\frac{1 + \frac{[H_2]}{K_i}}{1 + \frac{[H_2]}{K_b}} \right]$$

with:

$$\mathrm{E}_1^{0\mathrm{app}} = \mathrm{E}_1^{0\prime} - \frac{RT}{F} \times \frac{k_2 + k_{-2}}{k_2}$$

$$\mathrm{E}_2^{0\mathrm{app}} = \mathrm{E}_2^{0\prime} + \frac{RT}{F} \times \frac{k_2 + k_1}{k_2}$$

$$K_\mathrm{M} = \frac{k_2 + k_{-2}}{k'_{-1}}$$

$$K_i = \frac{k_2 + k_1}{k'_{-1}}$$

$$K_b = \frac{k_2}{k'_{-1}}$$

(Note that these parameters $E^0{}_1{}^{app}$, $E^0{}_2{}^{app}$ and K_M are not the same as in eqs 17, 26 and 27 in the main text.) The above relations imply the following constraints: $K_M > K_b$ and $K_i > K_b$.

Figure S2.2 shows how this model fits the experimental data (red and orange dashed line) and compares with the best fit obtained by assuming H_2 binding in chemical step 2 (black, same fit as that shown in figure 3 of the main text). The red lines are obtained fitting the limit case in which $K_b = K_M$ (0.3 mM in Cr HydA1 and 0.6 mM in Tam HydS), while the orange lines show the fit when K_b is forced to be smaller than K_M (K_b=0.05 mM in Cr HydA1 and K_b = 0.5 mM in Tam HydS). The limiting currents are fitted equally well by assuming H_2 binding in chemical step 1 and 2: the equation for $i_{lim}{}^{ox}$ is the same (the definition of K_M as a function of the rate constant changes), which allows the fitting of both models to precisely determine

the value of K_M. Assuming H_2 binding in step -1 renders the equation of i_{lim}^{red} more complex than having H_2 binding in step -2. The former model indeed predicts that the proton reduction activity is inhibited by H_2 with an inhibition constant K_i. FeFe-hydrogenases are known to be inhibited by H_2 but the inhibition constant is much greater than the H_2 concentration under saturating conditions. The parameter K_i was then fixed to 4 mM in Cr, as measured in ref 5, and 4 mM for Tam HydS as well.
The H_2 dependence of the E_{cat} is, instead, not reproduced by assuming H_2 binding in step k_{-1} (figure S2.2 panels A and B, orange and red curves). In particular, the model predicts an increase in E_{cat}^{ox} as a function of $[H_2]$ that is in contrast with the results of experiments, which confirms that H_2 binding happens in the chemical step 2 for both Cr HydA1 and Tam HydS. The same conclusion is reached assuming more complex kinetics of H_2 binding and release (SI section S2.3).

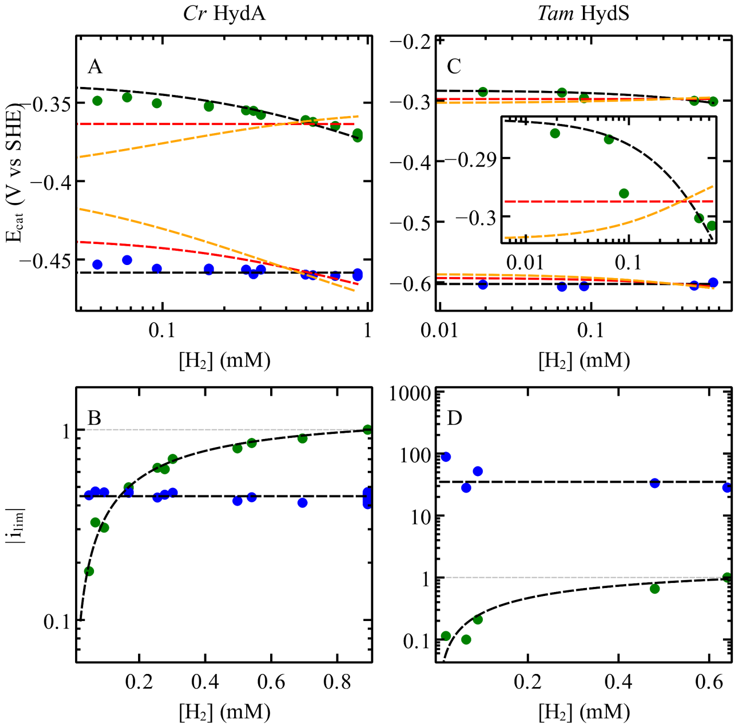

Figure S2.2 Fitting the $[H_2]$ pressure dependence of E_{cat} and I_{lim} for Cr HydA1 (panel A and B) and Tam HydS (panel C and D). The experimental data are the same as in figure 3 panels E, F, M and N (main text). E_{cat}^{ox} and I_{lim}^{ox} are shown in green, E_{cat}^{red} and I_{lim}^{red} in blue. The black dotted trace is the best fit obtained assuming unimolecular H_2 binding in step -2, as shown in figure 3 of the main text. The red and orange dashed lines are two fits of the model that assumes bimolecular H_2 binding in step -1 (the equations in section S2.2). The red line is the limit in which $K_b = K_M$ (0.3 mM for Cr HydA1 and 0.6 mM for Tam HydS), the orange line is the fit obtained having $K_b < K_M$ (Kb=0.05 mM for Cr HydA1 and K_b = 0.5 mM for Tam HydS). K_i was fixed to 4 mM for Cr HydA1, as measured in ref [5], and to 4 mM for Tam as well.

Note that this model for H_2 binding/release in chemical step 1, combined with the model of (de)protonation in chemical step 2 (section S2.1), are an approximation of model 2 in figure 1 of the main text, in which one only considers the cycle between active species (solid

arrows), forgetting about the inactivating branches. From the results in this section and in section S2.1 we conclude that assuming H_2 binding/release in the 1st chemical step and (de)protonation in the 2nd is not consistent with the experimental data for Cr HydA1 and Tam HydS.

Section S2.3 The kinetics of H_2 binding and release

In this section we describe a model for H_2 binding and release with more complex kinetics than that used in the main text. We consider a model similar to that proposed in ref [6], where the ligand diffuses through the enzyme and binds first close to the active site, to form a state that is called the geminate state, and then to the active site. Assuming that the diffusion step is fast compared to H_2 binding/release at the active site, we obtain the following expressions for the rate constants of hydrogen binding (k-) and release (k+):

$$k_+ = \frac{k_+^{\max}}{1 + \frac{[\mathrm{H_2}]}{K_\mathrm{G}}}$$

$$k_- = \frac{k_-^{\max}}{1 + \frac{K_\mathrm{G}}{[\mathrm{H_2}]}}$$

Where K_G is the dissociation constant of the H_2 in the geminate state.

Substitution of the above two equations in the definition of E_{cat} and I_{lim} (eqs 3-6 in the main text), and assuming that H_2 binding/release occurs in step 1 or 2 gives:

$$\mathrm{i}_{\mathrm{lim}}^{\mathrm{ox}} = \frac{\alpha}{1 + \frac{K_M}{[H_2]}}$$

$$\mathrm{i}_{\mathrm{lim}}^{\mathrm{red}} = \frac{\beta}{1 + \frac{[H_2]}{K_i}}$$

$$\mathrm{E}_{\mathrm{cat}}^{\mathrm{ox}} = \mathrm{E}_1^{0\mathrm{app}} + \frac{RT}{F} \ln \left[\frac{1 + \frac{[H_2]}{K_1}}{1 + \frac{[H_2]}{K_M}} \right]$$

$$\mathrm{E}_{\mathrm{cat}}^{\mathrm{red}} = \mathrm{E}_2^{0\mathrm{app}} + \frac{RT}{F} \ln \left[\frac{1 + \frac{[H_2]}{K_i}}{1 + \frac{[H_2]}{K_1}} \right]$$

with definitions of the parameters in Table S2.2. (Note that these parameters $E^0{}_1{}^{app}$, $E^0{}_2{}^{app}$ and K_M are not the same as in eqs 17, 26 and 27 in the main text.)

	Model 1 (H_2 binding/release in C1)	Model 2 (H_2 binding/release in C2)
E_1^{0app}	$E_1^{0\prime} + \frac{RT}{F} \ln \left[\frac{k_2}{k_2 + k_{-2}} \right]$	$E_1^{0\prime}$
E_2^{0app}	$E_2^{0\prime} + \frac{RT}{F} \ln \left[\frac{k_2 + k_1'}{k_2} \right]$	$E_2^{0\prime} + \frac{RT}{F} \ln \left[\frac{k_2 + k_1'}{k_2} \right]$
K_1	$K_G \times \frac{k_2}{k_2 + k'_{-1}}$	$K_G \times \frac{k_2' + k_{-1}}{k_{-1}}$
K_M	$K_G \times \frac{k_2 + k_{-2}}{k_2 + k_{-2} + k'_{-1}}$	$K_G \times \frac{k_2' + k_{-1}}{k'_{-2} + k_{-1}}$
K_i	$K_G \times \frac{k_2 + k_1'}{k_2 + k'_{-1}}$	$K_G \times \frac{k_2' + k_1 + k_{-1}}{k_1 + k_{-1}}$
α	$2FA\Gamma \frac{k_2 k'_{-1}}{k_2 + k_{-2} + k'_{-1}}$	$2FA\Gamma \frac{k'_{-2} k_{-1}}{k'_{-2} + k_{-1}}$
β	$-2FA\Gamma \frac{k_2 k_1'}{k_2 + k_1'}$	$-2FA\Gamma \frac{k_2' k_1}{k_2' + k_1 + k_{-1}}$
constraint	$K_1 < K_m$<br>$K_1 < K_i$	$K_1 > K_m$<br>$K_1 > K_i$

Table S2.2: Definition of the apparent potential and dissociation constant for H_2 binding/release in the first (model 1) or second (model 2) chemical step.

Figure S2.3 shows the best fits of the two models of table S2.2 to the experimental data shown in figure 3 panels E, F, M and N of the main text. Again the equations describing the limiting currents are the same for all the models and therefore fit equally well the data, allowing the measurement of K_M, the Michaelis constant, and K_i, the inhibition constant of proton reduction. As discussed in section S2.2 the value of K_i is high, and we therefore fixed it to 4 mM. Assuming this more complex kinetics of H_2 binding and release therefore does not change the conclusion drawn by simply assuming bimolecular H_2 binding and unimolecular H_2 release (main text and SI section S2.2). Indeed, only model 2, which assumes H_2 binding and release in chemical step 2, fits the data well (black dashed lines in figure S2.2). Model 1 was fitted in the limiting case in which $K_1 = K_M$ (red dashed lines) and when $K_1 < K_M$ (orange lines), and in both cases the H_2 dependence of the E_{cat} is not reproduced by the model, confirming that H_2 binding and release occur in the second chemical step.

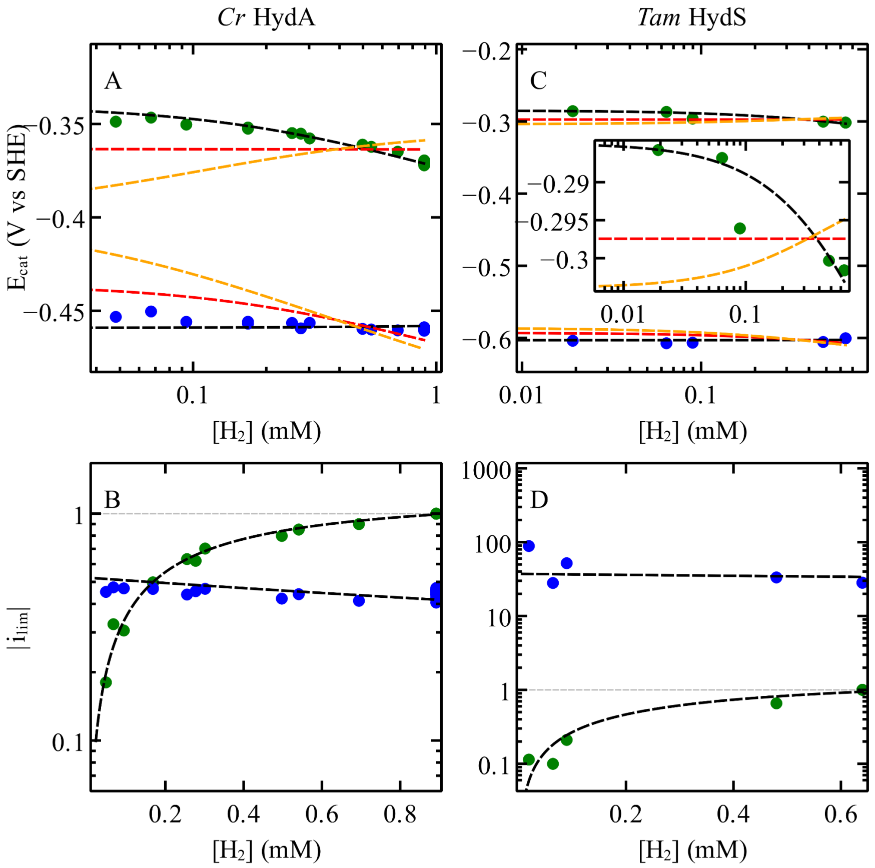

Figure S2.3 Fitting the H_2 pressure dependence of E_{cat} and I_{lim} for Cr HydA1 (panel A and B) and Tam HydS (panel C and D) considering the geminate state. E_{cat}^{ox} and I_{lim}^{ox} are shown in green, E_{cat}^{red} and I_{lim}^{red} in blue. The experimental data are the same as of figure 3 panels E, F, M and N. The black dash trace is the best fit obtained fitting model 2 (H_2 binding/release in the second chemical step). The red and orange dashed lines are obtained fitting model 1 (H_2 binding/release in the second chemical step). The red line is the limit in which $K_1 = K_M$ (0.3 mM for Cr HydA1 and 0.6 mM for Tam HydS), the orange line is the fit obtained having $K_1 < K_M$ (K_1=0.05 mM for Cr HydA1 and K_1=0.5 mM for Tam HydS). K_i was fixed to 4 mM for Cr HydA1, as measured in ref [5], and to 4 mM for Tam HydS as well. When fitting model 2 K_1 has to be larger than K_i and was therefore fixed to 5 mM in Cr HydA1 and 11 mM in Tam HydS.

Section S2.4 Relation between the acidity constant for the second protonation and K_3

The second protonation of the catalytic cycle occurs in the first chemical step and is the following reaction:

$$H_{sred}H^+ + H^+ \leftrightharpoons H_{hyd}H^+$$

Note that $H_{sred}H^+$ is only protonated once, while $H_{hyd}H^+$ is doubly protonated.
k_1 in the catalytic cycle is therefore the rate constant for the formation of $H_{hyd}H^+$ (protonation) and k_{-1} the rate constant for formation of $H_{sred}H^+$ (deprotonation).

The acidity constant K_a is therefore:

$$K_a = \frac{[\mathrm{H_{sred}H^+}] \times [\mathrm{H^+}]}{[\mathrm{H_{hyd}H^+}]} = \frac{k_{-1}}{k_1} \times [\mathrm{H^+}]$$

Introducing the kinetics of protonation defined in equations 9 and 10 of the main text, we obtain:

$$\frac{K_a}{[\mathrm{H^+}]} = \frac{k_{-1}^{max}}{1 + \frac{[\mathrm{H^+}]}{K_{relay}}} \times \frac{1 + \frac{K_{relay}}{[\mathrm{H^+}]}}{k_1^{max}}$$

which can be simplified in

$$K_a = \frac{k_{-1}^{max}}{k_1^{max}} \times K_{relay}$$

The definition of K_3 in equation 18 of the main text corresponds to K_a if k_2 is much smaller than the (de)protonation rate constants.

Section S2.5 Modeling of E_{cat} and i_{lim} as a function of pH at 5°C for Cr HydA1

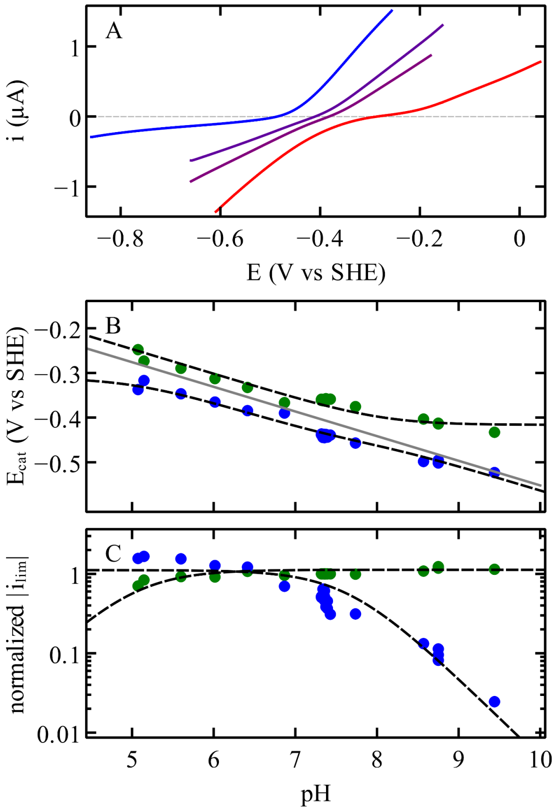

Figure S2.4: Cyclic voltammograms, catalytic potentials and limiting currents at different pHs for Cr HydA1 at 5°C The black dashed lines are the best fits of eqs 11-14 of the main text. The parameters are shown in Table S2.3. Panel A shows selected blank subtracted, averaged, cyclic voltammograms for Cr HydA1 at different pHs (at 1 atm [H_2], 5°C). Colors go from blue to red from high to low pH (pH values are 5.1, 6.9, 7.4, 8.8). Other conditions: scan rate 20 mV/s, electrode rotation rate 3000 rpm. The catalytic potentials

and the normalized limiting currents are plotted as a function of pH (panels B and C). The current values were normalized by the value of i_{lim}^{ox} at pH 7 in the plots of i_{lim} against pH. In green E_{cat}^{ox} and i_{lim}^{ox}, in blue E_{cat}^{red} and i_{lim}^{red}. In panel B a solid gray line indicates the Nernst potential of the H^+/H_2 couple.

	E^{0app}_1 (mV)	E^0_2 (mV)	pK	pK_1	pK_2	pK_3
Cr HydA1 5°C	-416	-457	8.1	< 5	< 5	7.7
Cr HydA1 30°C	-466	-523	8.3	< 5	< 5	7.9

Table S2.3: Parameters obtained by fitting eqs 11-14 (main text) to the data shown in figure S2.3, to interpret the pH-dependence of the catalytic potentials and limiting currents. The values at 30°C are repeated here to ease the comparison.

Section S2.6 Tam HydS K_M measurement

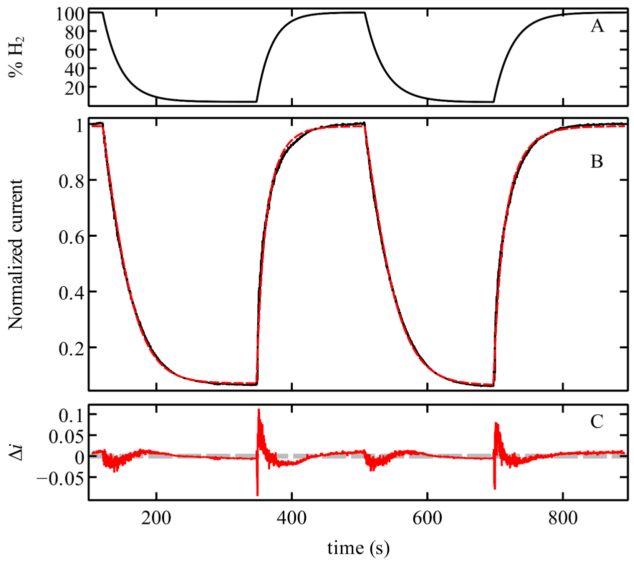

Figure S2.5: Measurement of the Michaelis constant of Tam HydS. Panel A shows the partial pressure of H_2 as a function of time in the experiment. Panel B shows the resulting catalytic current in solid black line and the fit of the Michaelis-Menten equation in dashed red line. The obtained value of K_M is 1 atm (which corresponds to 0.64 mM, considering the solubility of H_2 at 40°C). Panel C shows the difference between the data and the fit. Experimental conditions: -59 mV vs SHE; 40°C; pH 6.5; 3000 rpm.

Section S2.7 Modeling of the cyclic voltammograms of Cr HydA1 recorded at different pH values

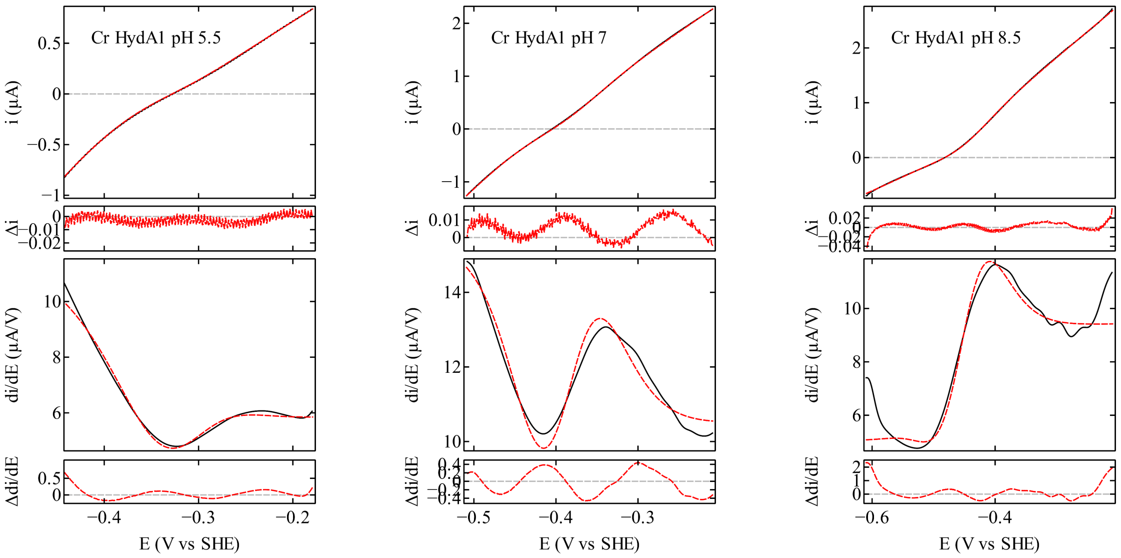

Figure S2.6: Fits of the generic EEC model to the voltammograms obtained with Cr HydA1 at pH 5.5, 7 and 8.5, from left to right. The top panel shows the experimental data in black solid lines and the fit in red dash lines. The residual (difference between data and fit) is shown in red in the panel below. The bottom panel shows the derivative of the data in black solid lines and the fit in dash red lines. Again, the residual is shown in the bottom panel. The capacitive current recorded without enzyme was subtracted from all the voltammograms. The 2 scans were then averaged. Conditions: scan rate 20 mV/s; 3000 rpm; at 30°C.

Section S2.8 Modeling of the voltammograms recorded with Cr HydA1 at different concentrations of H_2.

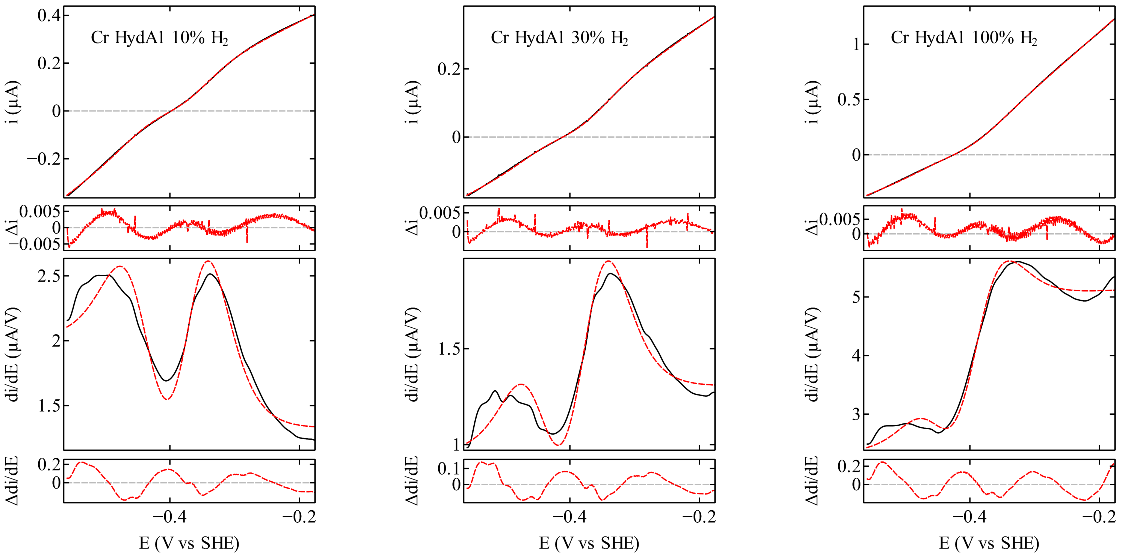

Figure S2.7: Fits of a generic EEC model to the voltammograms obtained with Cr HydA1 at pH 7.7, under 10, 30 and 100 % of H_2 from left to right. The top panel shows the experimental data in black solid lines and the fit in red dash lines. The residual (difference between data and fit) is shown in red in the panel below. The bottom panel shows the derivative of the data in black solid lines and the fit in dash red lines. Again the residual is shown in the panel below. The capacitive current recorded without enzyme was subtracted from all the voltammograms. The 2 scans were then averaged. Conditions: scan rate 20 mV/s; 3000 rpm; at 5°C.

Section S2.9 Modeling of the voltammograms recorded with Tam HydS at different concentrations of H_2

To analyse the voltammetry of Tam HydS at different H_2 pressures (figures S2.8, S2.9 and 2.10) one has to deal with the fact that the reductive current has much greater intensity than the oxidative current, especially at low H_2 partial pressure. To properly fit the model to the oxidative part of the voltammogram, we proceeded by analysing first the complete signal (panels A to D in figures S2.8, S2.9 and 2.10) to obtain the value of E_{cat}^{red}. The high potential part was then analyzed separately (panels E to H in figures S2.8, S2.9 and 2.10) to obtain the value of E_{cat}^{ox} as precisely as possible.

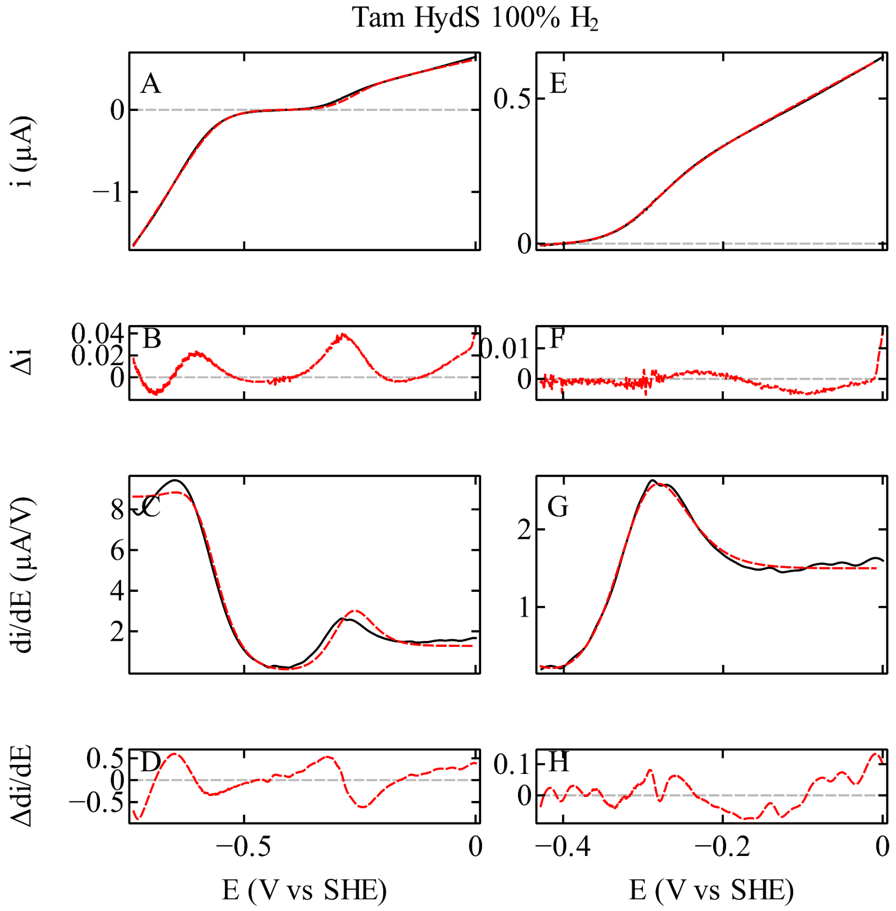

Figure S2.8: Fits of a generic EEC model to the voltammograms of Tam HydS at pH 6.5, under 100% H_2. The left column panels show the fit of the model to the entire CV, the right column panels show the analysis of the high potential part only. Panels A and E show the experimental data in black solid lines and the fit in red dash lines. The residual (difference between data and fit) is shown in red in the bottom panels (B and F). Panels C and G show the derivative of the data in black solid lines and the fit in dash red lines. Again, the residual is shown in the bottom panels (D and H). The capacitive current recorded without enzyme was subtracted from all the voltammograms. The 2 scans were then averaged. Conditions: scan rate 20 mV/s; 3000 rpm; at 40°C.

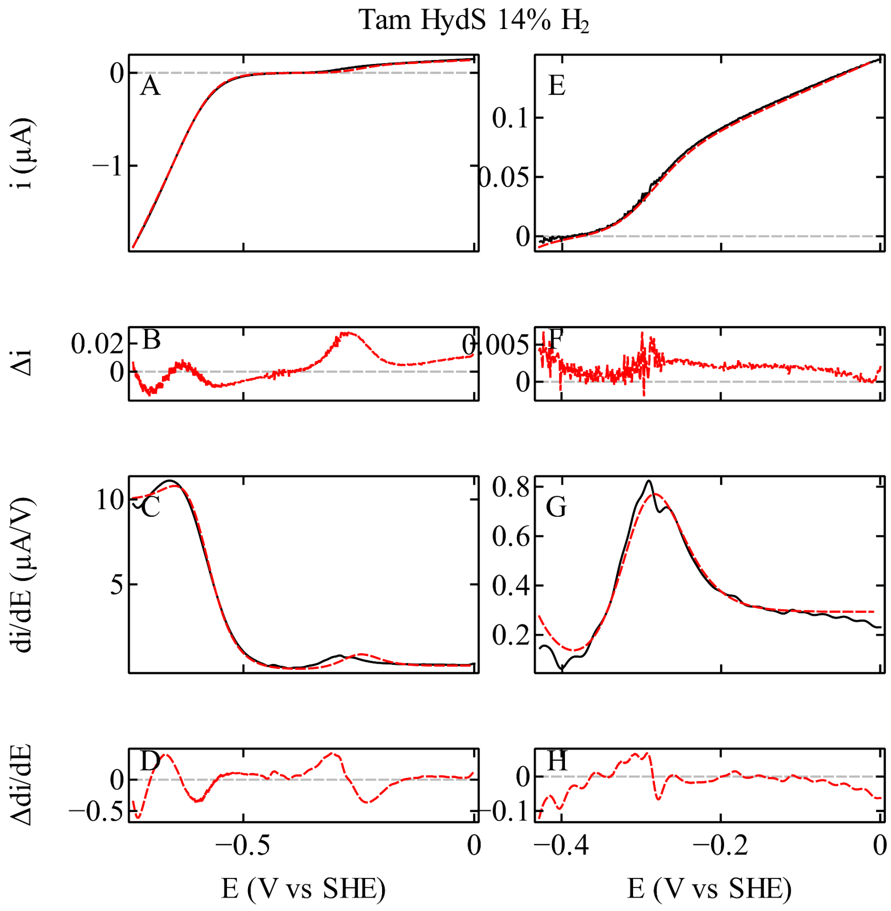

Figure S2.9: Fits of a generic EEC model to the voltammograms recorded with Tam HydS under pH 6.5, under 14% H_2 The left column panels show the fit of the model to the entire CV, the right column panels show the analysis of the high potential part only. Conditions: scan rate 20 mV/s; 3000 rpm; at 40°C.

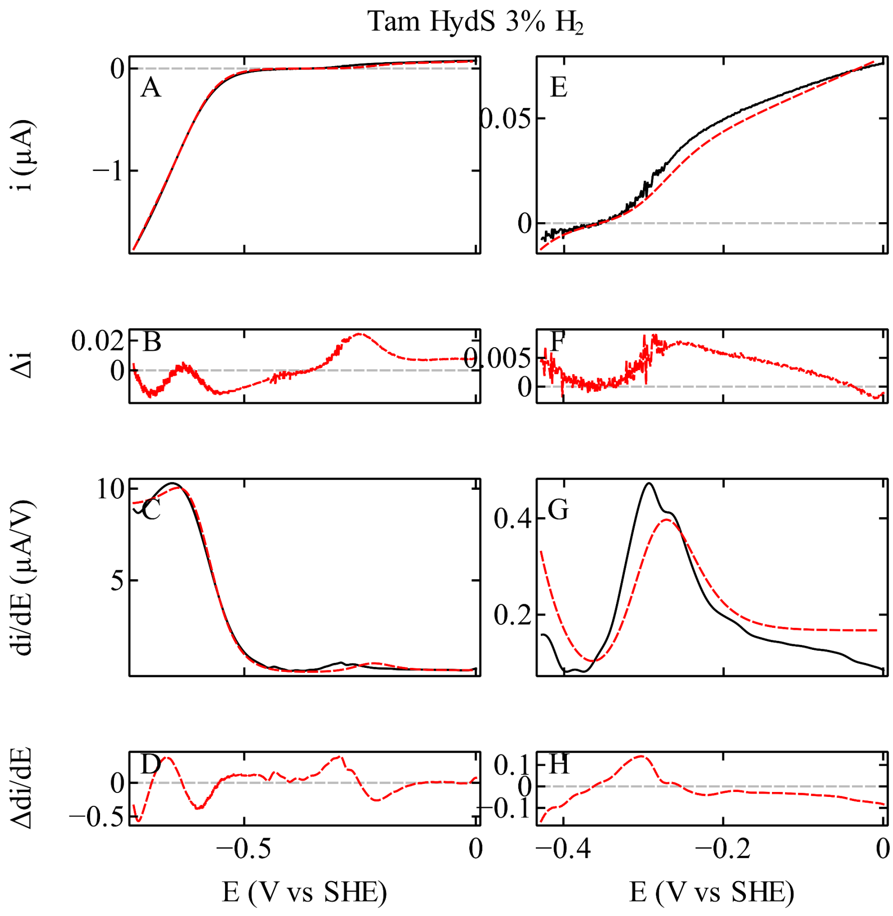

Figure S2.10: Fits of a generic EEC model to the voltammograms recorded with Tam HydS at pH 6.5, under 3% H_2 Conditions: scan rate 20 mV/s; 3000 rpm; at 40°C.

S2.10 Modeling the (de)protonation events for an ECEC kinetic scheme

The equation defining the catalytic potential and limited current in the case of an ECEC kinetic scheme were derived in ref [7] and copied below:

$$E_{\mathrm{cat}}^{\mathrm{ox}} = \frac{RT}{F}\ln\left[\frac{(k_1+k_{-1})\exp\frac{FE_0^1}{RT} + (k_2+k_{-2})\exp\frac{FE_0^2}{RT}}{k_{-1}+k_{-2}}\right]$$

$$E_{\mathrm{cat}}^{\mathrm{red}} = -\frac{RT}{F}\ln\left[\frac{(k_1+k_{-1})\exp\frac{-FE_0^2}{RT} + (k_2+k_{-2})\exp\frac{-FE_0^1}{RT}}{k_1+k_2}\right]$$

$$i_{\mathrm{lim}}^{\mathrm{ox}} = 2FA\Gamma\frac{k_{-1}k_{-2}}{k_{-1}+k_{-2}}$$

$$i_{\mathrm{lim}}^{\mathrm{red}} = -2FA\Gamma\frac{k_1k_2}{k_1+k_2}$$

Protonation kinetics such as that described by equations 9 and 10 of the main text was included in the 2 chemical steps, giving the following dependences of the catalytic potentials and limiting currents on pH:

$$E_{\mathrm{cat}}^{\mathrm{ox}} = E_0^{\mathrm{1app}} + \frac{RT}{F}\ln\left(1+\frac{[\mathrm{H}^+]}{K_1}\right)$$

$$E_{\mathrm{cat}}^{\mathrm{red}} = E_0^{\mathrm{2app}} + \frac{RT}{F}\ln\left(\frac{1}{1+\frac{K_1}{[\mathrm{H}^+]}}\right)$$

$$i_{\mathrm{lim}}^{\mathrm{ox}} = 2FA\Gamma\frac{\alpha}{1+\frac{[\mathrm{H}^+]}{K_{relay}}}$$

$$i_{\mathrm{lim}}^{\mathrm{ox}} = -2FA\Gamma\frac{\beta}{1+\frac{K_{relay}}{[\mathrm{H}^+]}}$$

It is clear that this model cannot fit the experimental data shown in figure 3 (panels B, C, H and I): the limiting currents should both decrease 1 decade per pH unit above ($i_{\mathrm{lim}}^{\mathrm{red}}$) or below ($i_{\mathrm{lim}}^{\mathrm{ox}}$) the value of pK_{relay}, which is not something we see in the experimental data; $E_{\mathrm{cat}}^{\mathrm{ox}}$ should increase with a slope of -60 mV/pH at pH below pK_1 while $E_{\mathrm{cat}}^{\mathrm{red}}$ should decrease 60 mV/pH at pH above pK_1, again not observed in the experimental data, no matter the value of pK_1.

Below are the definition of the parameters of the previous equations:

$$E_0^{\mathrm{1app}} = \frac{E_0^1 + \frac{RT}{F}\ln(k_{-1}^{\mathrm{max}}) + E_0^2 + \frac{RT}{F}\ln(k_{-2}^{\mathrm{max}})}{k_{-1}^{\mathrm{max}} + k_{-2}^{\mathrm{max}}}$$

$$E_0^{2\mathrm{app}} = \frac{\left[E_0^1 + \frac{RT}{F}\ln(k_1^{\max} + k_2^{\max})\right] \times \left[E_0^2 + \frac{RT}{F}\ln(k_1^{\max} + k_2^{\max})\right]}{E_0^1 + \frac{RT}{F}\ln(k_1^{\max}) + E_0^2 + \frac{RT}{F}\ln(k_2^{\max})}$$

$$K_1 = K_{\mathrm{relay}} \times \frac{E_0^1 + \frac{RT}{F}\ln(k_{-1}^{\max}) + E_0^2 + \frac{RT}{F}\ln(k_{-2}^{\max})}{E_0^1 + \frac{RT}{F}\ln(k_1^{\max}) + E_0^2 + \frac{RT}{F}\ln(k_2^{\max})}$$

$$\alpha = \frac{k_{-2}^{\max} k_{-1}^{\max}}{k_{-2}^{\max} + k_{-1}^{\max}}$$

$$\beta = \frac{k_2^{\max} k_1^{\max}}{k_2^{\max} + k_1^{\max}}$$

Section S3. Preparation of [2Fe]ADT- activated TamHydS

The preparation of the holo-form of TamHydS was performed as previously reported with minor changes to the procedure.[4] For the expression of the apo-form, sequence-confirmed plasmids were transformed in chemically competent E. coli BL21(DE3) cells. The cells were cultivated in sterile M9 medium (22 mM KH_2PO_4, 22 mM Na_2HPO_4, 18 mM NH_4Cl, 0.2 mM $MgSO_4$, 85 mM NaCl, 0.1 mM $CaCl_2$, 4 g L^{-1} D-glucose) at 37 °C. After reaching O.D.600 ≈ 0.5, the cultivation temperature was lowered to 20°C, and the protein expression was induced with 1 mM IPTG with concomitant supplementation of the culture with 100 µM $FeSO_4$ in 1% HCl solution. The cell pellet was harvest ≈ 16 h after induction and stored at −20 °C until lysis. The cell lysis, the protein purification, the reconstitution of the [4Fe-4S] clusters, as well as the activation of the enzyme were carried out in an MBRAUN glovebox under argon atmosphere (app. 1 ppm O_2). The cell were lysed by resuspending the pellet in lysis buffer (10 mM $MgCl_2$, 10 mg mL^{-1} lysozyme, 0.05 mg mL^{-1} RNAse and 0.05 mg mL^{-1} DNAse in 100 mM Tris-HCl, 150 mM NaCl pH 8.0) and a subsequent sonication. The protein was purified using StrepTrap affinity chromatography (StrepTrap HP (GE Healthcare)) following the manufacturer's instructions, with an additional washing step using a sub-denaturing concentration of 1 M urea in 100 mM Tris-HCl, 150 mM NaCl pH 8.0.[8,9] A subsequent size-exclusion chromatography (Superdex 200 HP (GE Healthcare)) step was performed for the removal of low molecular weight impurities. After the two purification steps, the protein yield was 0.6 mg L^{-1} of cell culture with an iron/protein content of 7 Fe/protein. By incubating the enzyme (50 µM) in a reaction with 500 µM dithiothreitol, 500 nM cysteine desulferase (*E. coli* IscS), 700 µM L-cysteine and 700 µM $(NH_4)_2Fe(SO_4)_2(H_2O)_6$ in 100 mM Tris-HCl, 150 mM NaCl pH 8.0 all [4Fe-4S] clusters were fully reconstituted (16.8 Fe/protein). Subsequently, the apo-form of TamHydS (50 µM) was activated by incubating it for 2 h with 1 mM sodium dithionite and 600 µM [2Fe]ADT in 100 mM phosphate buffer pH 6.8, and desalted with 10 mM Tris-HCl pH 8.0. After concentrating the generated holo-form of TamHydS, aliquots were prepared in air-tight vials, flash frozen in liquid N_2 and stored at −80 °C. The successful activation with [2Fe]ADT and the correct cofactor integration were verified by ATR-FTIR, and EPR analyses.

References

(1) Sommer, C.; Adamska-Venkatesh, A.; Pawlak, K.; Birrell, J. A.; Rüdiger, O.; Reijerse, E. J.; Lubitz, W. Proton Coupled Electronic Rearrangement within the H-Cluster as an Essential Step in the Catalytic Cycle of [FeFe] Hydrogenases. *J. Am. Chem. Soc.* **2017**, *139* (4), 1440–1443. doi: 10.1021/jacs.6b12636

(2) Birrell, J. A.; Rodríguez-Maciá, P.; Hery-Barranco, A. A Beginner's Guide to Thermodynamic Modelling of [FeFe] Hydrogenase. *Catalysts* **2021**, *11* (2), 238. doi: 10.3390/catal11020238

(3) Laun, K.; Baranova, I.; Duan, J.; Kertess, L.; Wittkamp, F.; Apfel, U.-P.; Happe, T.; Senger, M.; Stripp, S. T. Site-Selective Protonation of the One-Electron Reduced Cofactor in [FeFe]-Hydrogenase. *Dalton Trans.* **2021**, *50* (10), 3641–3650. doi: 10.1039/d1dt00110h

(4) Land, H.; Sekretareva, A.; Huang, P.; Redman, H. J.; Németh, B.; Polidori, N.; Mészáros, L. S.; Senger, M.; Stripp, S. T.; Berggren, G. Characterization of a Putative Sensory [FeFe]-Hydrogenase Provides New Insight into the Role of the Active Site Architecture. *Chem. Sci.* **2020**, *11* (47), 12789–12801. doi: 10.1039/d0sc03319g

(5) Fourmond, V.; Baffert, C.; Sybirna, K.; Dementin, S.; Abou-Hamdan, A.; Meynial-Salles, I.; Soucaille, P.; Bottin, H.; Léger, C. The Mechanism of Inhibition by H2 of H2-Evolution by Hydrogenases. *Chem. Commun.* **2013**, *49* (61), 6840–6842. doi: 10.1039/c3cc43297a

(6) Liebgott, P.-P.; Leroux, F.; Burlat, B.; Dementin, S.; Baffert, C.; Lautier, T.; Fourmond, V.; Ceccaldi, P.; Cavazza, C.; Meynial-Salles, I.; Soucaille, P.; Fontecilla-Camps, J. C.; Guigliarelli, B.; Bertrand, P.; Rousset, M.; Léger, C. Relating Diffusion along the Substrate Tunnel and Oxygen Sensitivity in Hydrogenase. *Nat. Chem. Biol.* **2010**, *6* (1), 63–70. doi: 10.1038/nchembio.276

(7) Fourmond, V.; Wiedner, E. S.; Shaw, W. J.; Léger, C. Understanding and Design of Bidirectional and Reversible Catalysts of Multielectron, Multistep Reactions. *J. Am. Chem. Soc.* **2019**, *141* (28), 11269–11285. doi: 10.1021/jacs.9b04854

(8) Belval, L.; Marquette, A.; Mestre, P.; Piron, M.-C.; Demangeat, G.; Merdinoglu, D.; Chich, J.-F. A Fast and Simple Method to Eliminate Cpn60 from Functional Recombinant Proteins Produced by E. Coli Arctic Express. *Protein Expr. Purif.* **2015**, *109*, 29–34. doi: 10.1016/j.pep.2015.01.009

(9) Cabotaje, P. R.; Walter, K.; Zamader, A.; Huang, P.; Ho, F.; Land, H.; Senger, M.; Berggren, G. Probing Substrate Transport Effects on Enzymatic Hydrogen Catalysis: An Alternative Proton Transfer Pathway in Putatively Sensory [FeFe] Hydrogenase. *ACS Catal.* **2023**, *13* (15), 10435–10446. doi: 10.1021/acscatal.3c02314
